# Interorgan Crosstalk in MASLD: A Narrative Review

**DOI:** 10.3390/biomedicines14091949

**Published:** 2026-08-29

**Authors:** Amedeo Lonardo, Ralf Weiskirchen

**Affiliations:** 1 Independent Researcher, 41100 Modena, Italy; 2 Institute of Molecular Pathobiochemistry, Experimental Gene Therapy and Clinical Chemistry (IFMPEGKC), RWTH University Hospital Aachen, D-52074 Aachen, Germany; rweiskirchen@ukaachen.de

**Keywords:** gut microbiome, insulin resistance, bile acids and salts, extracellular vesicles, intercellular signaling peptides and proteins, MASLD

## Abstract

Metabolic dysfunction-associated steatotic liver disease (MASLD) is a systemic disorder shaped by interorgan crosstalk: dynamic, bidirectional communication through which the liver and endocrine organs, gut, adipose tissue, brain, kidney, skeletal muscle, bone, and heart exchange signals to coordinate metabolism, immunity, and tissue homeostasis. Across these axes, neural circuits, hormones, cytokines, adipokines, hepatokines, myokines, osteokines, bile acids, microbial metabolites, lipids, extracellular vesicles, and microRNAs integrate nutrient handling, insulin action, immunity, mitochondrial function, and tissue remodeling. Perturbation of these networks converts physiological homeostasis into self-reinforcing loops of substrate overflow, endocrine dysregulation, dysbiosis, inflammation, and fibrogenesis, while hepatic dysfunction propagates renal, neurocognitive, cardiometabolic, and musculoskeletal complications. This framework helps explain why individuals with comparable steatosis show divergent trajectories of metabolic dysfunction-associated steatohepatitis (MASH), fibrosis, extrahepatic disease, and treatment response. It also highlights tractable points of intervention, including restoration of adipose buffering, modulation of gut microbial and bile-acid signaling, correction of endocrine drivers, preservation of muscle and bone, and integrated cardio–kidney–liver risk reduction across different disease stages and clinical phenotypes. We argue that precision hepatology should move beyond isolated assessment of liver fat and fibrosis towards multidimensional phenotyping of dominant crosstalk mechanisms. Longitudinal multi-omic studies and trials incorporating outcomes across organs are now required to distinguish causal signals from disease correlates, define clinically actionable endotypes, and test whether targeting one node can restore durable metabolic and functional resilience throughout the interconnected MASLD network, while improving patient-centered outcomes across the disease course.

## 1. Introduction

Functional organ axes are dynamic, bidirectional networks that coordinate organ function and maintain homeostasis [1,2,3,4]. Molecular and cellular signaling integrates organ-specific responses, and its disruption causes disease. For example, the brain regulates gut motility, while the gut conveys nutrient status to central pathways [5,6]. These axes link the digestive, nervous, endocrine, and immune systems through hormones, autonomic pathways, cytokines, and microbial metabolites [7]. Their disruption can cause metabolic and inflammatory diseases [8]. Functional medicine uses this framework to diagnose and manage multisystem disorders [9].

Metabolic dysfunction-associated steatotic liver disease (MASLD), formerly nonalcoholic fatty liver disease (NAFLD), is a major multisystem disorder defined by hepatic steatosis with cardiometabolic risk [10]. Its spectrum extends from steatosis associated with metabolic dysfunction to metabolic dysfunction-associated steatohepatitis (MASH), progressive fibrosis, cirrhosis, and HCC [10]. Prevalence is increasing worldwide, with substantial geographical variation and particularly high estimates in parts of the Middle East [11]. Outcomes nevertheless extend beyond liver-related events to CVD, chronic kidney disease (CKD), endocrine dysfunction, neurocognitive impairment and loss of musculoskeletal resilience.

This clinical breadth is incompatible with a model of the liver as an isolated lipid repository. Homeostasis depends on dynamic, bidirectional axes linking the liver with the brain, gut, endocrine organs, adipose tissue, kidney, muscle and bone [1,2,3,4,5,6,7].

Neural circuits, hormones, cytokines, microbial metabolites, bile acids, lipids, microRNAs and extracellular vesicles coordinate energy balance, insulin action, immunity and stress adaptation. When these networks fail, physiological signaling becomes a self-reinforcing driver of substrate overflow, insulin resistance, inflammation, mitochondrial dysfunction and tissue injury [8].

Adipose dysfunction increases fatty-acid flux and inflammation; endocrine disturbances impair lipid handling and mitochondrial function; gut dysbiosis and barrier failure alter portal microbial and bile-acid signaling; and reduced muscle glucose disposal worsens substrate excess. In turn, the steatotic, fibrotic liver releases metabolites, lipoproteins, hepatokines, cytokines and extracellular vesicles that disrupt distant tissues. These reciprocal loops help explain divergent inflammation, fibrosis, complications and treatment responses despite similar steatosis.

A systems perspective shifts assessment from liver fat to the dominant disease-sustaining circuits. Treatment may combine restoration of adipose buffering, correction of endocrine drivers, modulation of gut microbial and bile-acid signaling, preservation of muscle and bone, integrated cardio–kidney–liver risk reduction and liver-directed therapy. This review synthesizes the endocrine–liver, gut–liver, adipose tissue–liver, liver–brain, liver–kidney and muscle–bone axes, examining how neural, hormonal, immune, microbial, metabolic and vesicular signals shape disease and reveal targets for mechanistic stratification and intervention.

This review presents a bidirectional, stage-dependent model of MASLD that integrates endocrine, gut, adipose, brain, kidney, muscle, bone, and heart axes. Linking shared mechanisms to divergent trajectories, endotypes and cross-organ treatment effects advances dynamic network-based stratification and supports multidimensional phenotyping, cross-organ trial endpoints and combination therapies that preserve systemic resilience beyond liver histology.

## 2. Methods

This narrative review synthesizes current concepts of interorgan communication in MASLD, focusing on the endocrine–liver, gut–liver, adipose tissue–liver, liver–brain, liver–kidney, MASLD–muscle–bone and MASLD-heart axes. MASLD was defined according to current nomenclature as hepatic steatosis with at least one cardiometabolic risk factor, without harmful alcohol consumption or another predominant cause of steatosis. Given the heterogeneity of experimental, epidemiological, clinical, multi-omic and therapeutic evidence across organ systems, we adopted a mechanistic and translational narrative approach rather than a systematic review or meta-analysis.

Relevant articles were identified through targeted searches of PubMed/MEDLINE, Web of Science, Scopus, and Google Scholar, supplemented by manual screening of reference lists from key reviews, guidelines, mechanistic studies, and landmark clinical trials.

Searches combined terms related to MASLD and its previous nomenclature with terms describing organ crosstalk and individual axes, including “metabolic dysfunction-associated steatotic liver disease,” “MASLD,” “NAFLD,” “MASH,” “organ crosstalk,” “interorgan communication,” “endocrine–liver axis,” “gut–liver axis,” “adipose tissue–liver axis,” “liver–brain axis,” “liver–kidney axis,” “sarcopenia,” “myosteatosis,” “osteoporosis,” “extracellular vesicles,” “exosomes,” “microRNA,” “bile acids,” “hepatokines,” “myokines,” “adipokines,” and “gut microbiota.” Priority was given to articles published in English, with an emphasis on recent publications, international guidelines, high-impact reviews, meta-analyses, prospective cohort studies, translational studies, and mechanistic experimental work relevant to MASLD pathogenesis, progression, heterogeneity, and treatment responsiveness. Older seminal studies were included when they provided foundational concepts for functional organ axes, neuroendocrine regulation, gut-derived signaling, adipose inflammation, insulin resistance, hepatic encephalopathy, CKD, sarcopenia, bone metabolism, or extracellular-vesicle biology. Studies were considered eligible when they addressed bidirectional or functionally relevant communication between the liver and at least one extrahepatic organ system, or when they clarified molecular mediators such as hormones, cytokines, adipokines, hepatokines, myokines, osteokines, bile acids, microbial metabolites, extracellular vesicles, microRNAs, lipid intermediates, or neural pathways. Articles focused exclusively on isolated hepatic lipid metabolism without relevance to systemic signaling, extrahepatic disease, or therapeutic implications were considered of lower priority.

Searches were conducted for studies published from January 2000 to June 2026. The evidence was organized into thematic sections reflecting major biological axes rather than according to study design, because MASLD is increasingly understood as a multisystem disorder embedded within dynamic endocrine, metabolic, immune, neural, microbial, renal, musculoskeletal, and adipose networks. For each axis, we extracted and synthesized information on anatomical and physiological foundations, principal molecular mediators, mechanisms contributing to steatosis, inflammation, fibrogenesis, extrahepatic complications, clinical heterogeneity, and therapeutic opportunities. Attention was paid to reciprocal signaling loops, because several axes operate bidirectionally, with the liver acting both as a recipient of pathogenic extrahepatic signals and as an active endocrine, metabolic, inflammatory, and hemodynamic organ influencing distant tissues. Where available, clinical evidence was integrated with mechanistic data to distinguish associations from plausible causal pathways and to identify areas in which experimental evidence remains stronger than human validation. Because terminology has evolved from nonalcoholic fatty liver disease (NAFLD) and nonalcoholic steatohepatitis (NASH) to MASLD and MASH, older studies using previous nomenclature were retained when their populations and metabolic phenotypes were conceptually applicable to MASLD, while acknowledging that definitions are not invariably interchangeable. Study selection and interpretation were performed by both authors, and disagreements regarding inclusion, strength of evidence, or causal wording were resolved by discussion and consensus. The final synthesis was developed through iterative discussion among the authors, with the aim of producing an integrative framework that connects organ-specific mechanisms with precision risk stratification and mechanism-based therapeutic development in MASLD.

There are notable differences between the present clinically oriented review article and a previous publication on the same topic by Yang et al. [8]. Whereas earlier work catalogued molecular mediators, this review reconceptualizes MASLD as a dynamic, bidirectional network linking hepatic, endocrine, intestinal, adipose, neural, renal, cardiovascular and musculoskeletal systems. By connecting organ crosstalk to disease heterogeneity, evidential maturity and cross-organ therapeutics, the present article advances mechanism-based endotyping, integrated risk stratification and patient-centered precision hepatology.

## 3. The Endocrine–Liver Axis in MASLD

The liver both responds to endocrine signals and mediates hormone synthesis, biotransformation, and clearance [12]. Disrupted neuroendocrine and glandular circuits can therefore create bidirectional feedback loops that promote steatosis and inflammation [13]. MASLD is thus not solely driven by caloric excess and insulin resistance [14] but is embedded in endocrine and metabolic networks [15,16,17]. This section examines the hypothalamus–pituitary, pancreas–liver, thyroid–liver and gonad–liver axes.

### 3.1. The Hypothalamus–Pituitary Axis

The hypothalamus integrates neural and hormonal cues to coordinate appetite, energy expenditure and peripheral metabolism [18]. Disruption of hypothalamic–pituitary signaling is an established experimental cause of liver injury, and progressive MASLD/MASH is observed after neurosurgery for craniopharyngioma [19].

#### 3.1.1. Growth Hormone Deficiency and Steatotic Liver Disease

Growth hormone (GH) stimulates hepatic insulin-like growth factor-1 (IGF-1) production, primarily through Janus kinase 2–STAT5 signaling [20]. Their metabolic effects, however, differ. GH is counter-regulatory: it promotes lipolysis, raises circulating free fatty acids, and can impair hepatic and skeletal-muscle insulin sensitivity through the Randle cycle, consistent with insulin resistance in acromegaly and possible hyperglycemia during GH replacement. In contrast, IGF-1 is chiefly insulin-sensitizing and may reduce inflammation and redirect nutrients away from hepatic storage. GH may directly reduce hepatic steatosis by suppressing de novo lipogenesis, enhancing β-oxidation, and activating STAT5-dependent detoxification pathways, whereas sustained GH/IGF-1 signaling may limit inflammation and IGF-1 may attenuate fibrosis by inhibiting hepatic stellate-cell activation [20]. Therefore, GH can lower liver fat despite worsening systemic insulin sensitivity, while the insulin-sensitizing and antifibrotic effects of the axis are largely attributable to IGF-1 [20].

The relationship between this axis and MASLD is bidirectional. A meta-analysis of 18 studies comprising 20,520 middle-aged participants found inverse associations between circulating GH or IGF-1 and both the presence and severity of MASLD [21].

In adults with GH deficiency or panhypopituitarism, loss of this hepatoprotective axis is associated with rapid progression from steatosis to advanced MASH. Low IGF-1 might also facilitate fibrosis through direct effects on stellate-cell activation [22]. Although evidence remains limited, recombinant GH or GH-releasing hormone analogues can reduce liver fat and might slow fibrosis [22].

#### 3.1.2. Prolactin and Adrenocorticotropic Alterations

Prolactin (PRL), secreted by the anterior pituitary, is emerging as a regulator of metabolic homeostasis and liver health [23].

Very high (>100 µg/L) and very low (<7 µg/L) PRL concentrations are associated with adverse outcomes, whereas moderately elevated concentrations within or just above the physiological range—termed HomeoFIT-PRL—seem metabolically favorable. In experimental models and humans, HomeoFIT-PRL improves insulin resistance, glucose tolerance, adipose hypertrophy and SLD through coordinated actions in the pancreas, liver, adipose tissue and hypothalamus [24].

PRL also activates hypothalamic proopiomelanocortin neurons, increasing hepatic sympathetic outflow and suppressing fatty-acid synthase (FAS)-dependent lipogenesis [25]. This PRL-dependent brain–liver circuit is a potential therapeutic target in MASLD.

By contrast, hypothalamic–pituitary–adrenal dysregulation increases adrenocorticotropic hormone and cortisol exposure. Chronic subclinical hypercortisolism promotes visceral adiposity and free-fatty-acid delivery to the liver, thereby increasing triglyceride synthesis [26].

In transgenic mice, corticosterone-induced expression of hepatic lipid-metabolism proteins contributes to high-fat-diet-induced MASLD [27].

In humans, MASLD is more prevalent in Cushing’s syndrome than in controls, with risk determined mainly by metabolic burden, disease duration and etiology [28,29]. SLD may regress only partly after remission, although fibrosis risk declines most clearly in patients with high baseline fibrosis. These data support systematic liver assessment in Cushing’s syndrome, particularly after prolonged disease exposure or in patients with substantial metabolic risk [28].

### 3.2. The Pancreas–Liver Axis

Pancreas–liver crosstalk is central to MASLD: pancreatic insulin regulates systemic glucose and lipid metabolism, whereas the liver determines insulin sensitivity and clearance.

#### 3.2.1. Molecular Mechanisms of Hepatic Insulin Resistance in Steatosis and De Novo Lipogenesis

Hepatic insulin resistance arises when lipid excess disrupts insulin signaling while preserving lipogenesis [30,31]. Free fatty acids promote diacylglycerol and ceramide accumulation, inflammation, and mitochondrial reactive oxygen species [30]. Membrane diacylglycerol activates protein kinase C epsilon, which phosphorylates and inhibits the insulin receptor, reducing insulin receptor substrate 1/2–phosphoinositide 3-kinase–protein kinase B signaling. Ceramides additionally activate atypical protein kinase C zeta or protein phosphatase 2A, further suppressing protein kinase B [30]. Consequently, insulin inadequately restrains hepatic glucose production and gluconeogenesis. Nevertheless, hyperinsulinemia can sustain sterol regulatory element-binding protein 1c (SREBP-1c)-driven de novo lipogenesis, producing selective insulin resistance [30]. Mechanistic divergence occurs downstream of protein kinase B: mechanistic target of rapamycin complex 1 and ribosomal protein S6 kinase 1 promote lipogenesis and inhibitory insulin receptor substrate phosphorylation, whereas atypical protein kinase C maintains SREBP-1c and nuclear factor kappa B activity. Hepatic zonation and distinct insulin receptor substrate isoforms may reinforce this uncoupling of glucose and lipid metabolism [30]. Hyperinsulinemia activates SREBP-1c and carbohydrate response element-binding protein (ChREBP) [31], increasing DNL and hepatic triglyceride accumulation [32].

The widely cited concept of selective hepatic insulin resistance remains debated. Although disruption of INSR–IRS–PI3K–AKT signaling can suppress de novo lipogenesis, circulating fatty acids, glucose-derived substrates, and extrahepatic insulin resistance also drive hepatic fat accumulation. Thus, as discussed under Section 5.2, MASLD reflects interacting hepatic and peripheral pathways rather than a single preserved lipogenic signaling mechanism, with contributions varying across contexts.

#### 3.2.2. Defective Insulin Clearance via Carcinoembryonic Antigen-Related Cell Adhesion Molecule 1

Hepatic insulin clearance depends partly on carcinoembryonic antigen-related cell adhesion molecule 1 (CEACAM1), which mediates internalization and degradation of the insulin-receptor complex. Nutrient excess suppresses CEACAM1, sustaining hyperinsulinemia, systemic insulin resistance, lipid flux, and inflammatory signaling [33].

#### 3.2.3. The Mutual and Bidirectional Relationship Between Type 2 Diabetes (T2D) and MASLD

T2D is a major determinant of adverse liver outcomes: more than 70% of affected individuals have MASLD, and up to 30% develop MASH, with greater inflammation and fibrosis. Conversely, MASLD impairs glycemic control and accelerates pancreatic β-cell failure [34].

T2D independently increases the risk of liver-stiffness progression and liver-related events in MASLD. Adequate glycemic control attenuates stiffness progression, although clear effects on regression or liver-related events remain unproven [35].

### 3.3. The Thyroid–Liver Axis

The thyroid–liver axis integrates thyroid hormone signaling with hepatic lipid handling, mitochondrial function, and cholesterol metabolism [36].

The liver is both a target and a component of the hypothalamic–pituitary–thyroid axis: it produces thyroid hormone-binding proteins and expresses deiodinases that convert thyroxine (T_4_) to triiodothyronine (T_3_), thereby shaping local and systemic hormone availability [37].

#### 3.3.1. Thyroid Hormone Receptor Beta Signaling

Most hepatic effects of T_3_ are mediated by thyroid hormone receptor-β (THR-β), the predominant hepatocyte isoform. THR-β promotes mitochondrial biogenesis and fatty-acid oxidation, partly through carnitine palmitoyltransferase 1A, and supports autophagic disposal of lipotoxic substrates [38]. It also increases low-density lipoprotein (LDL) receptor expression, linking thyroid signaling to cholesterol clearance [39].

#### 3.3.2. Hypothyroidism and TSH Pathogenicity

Both overt and subclinical hypothyroidism are enriched in MASLD, supporting a clinically relevant association between impaired thyroid homeostasis and metabolic liver disease [40,41].

Reduced thyroid hormone availability is accompanied by lower hepatic THR-β expression, particularly in advanced steatosis. Impaired receptor signaling reduces mitochondrial fatty-acid oxidation and favors accumulation of unesterified fatty acids, reinforcing the steatogenic process and hepatocellular lipotoxicity in a subset of individuals [42].

Hepatocytes also express functional thyroid-stimulating hormone receptors (TSH-R) [43]. Elevated TSH can therefore act directly on the liver, independently of thyroid hormone availability [44]. TSH-R engagement activates cAMP signaling, increases SREBP-1c activity, and suppresses AMP-activated protein kinase (AMPK), shifting metabolism towards lipogenesis [45]. Collectively, data suggest that although TSH is not directly hepatotoxic, TSH-R activation is a key requisite for promoting hepatic steatogenesis.

These mechanisms motivated the development of liver-directed selective THR-β agonists, designed to reduce hepatic lipid accumulation and improve MASH histology while limiting extrahepatic T_3_-related effects [46].

Because MASH can progress to HCC, preclinical evidence that resmetirom suppresses midkine–LDL receptor-related protein 1-mediated immunosuppressive crosstalk provides a rationale for investigating whether this approved MASH therapy can also limit hepatocarcinogenesis [47].

### 3.4. The Gonad–Liver Axis

Gonadal signaling contributes to MASLD pathobiology and its marked sexual dimorphism [48,49,50,51]. Premenopausal women are relatively protected from severe steatosis and fibrosis compared with age-matched men, but this advantage diminishes after menopause, consistent with loss of estrogen-dependent hepatometabolic protection [48].

The high prevalence and rapid progression of liver disease in women with polyendocrine metabolic ovary syndrome (PMOS), previously termed polycystic ovary syndrome (PCOS), further implicate sex hormones in liver health [48,49].

#### 3.4.1. The Ovary–Liver Axis in MASLD

In women, ovarian endocrine function modifies MASLD risk through effects on adipose distribution, glucose–lipid homeostasis and hepatic inflammation. This ovary–liver axis is driven mainly by estradiol acting through nuclear estrogen receptors and membrane-initiated pathways [50].

Endogenous estrogens, particularly 17β-estradiol, exert antisteatotic, anti-inflammatory and antifibrotic actions, principally through hepatic ER-α. ER-α suppresses lipogenic genes including *SREBP1c*, *FASN* and acetyl-CoA carboxylase (*ACC*), whereas ER-α/ER-β programs promote lipid oxidation. G protein-coupled estrogen receptor signaling also regulates glucose uptake, mitochondrial function and inflammation through rapid phosphoinositide 3-kinase (PI3K)–MAPK pathways [51].

Estrogen signaling preserves mitochondrial integrity, limits reactive oxygen species and restrains Kupffer-cell activation. Menopause or ovarian dysfunction therefore removes an important hepatometabolic brake, favoring triglyceride accumulation, cytokine activation, steatohepatitis and collagen deposition [50,51].

#### 3.4.2. Androgenic Dichotomy: Testosterone and PMOS

Testosterone exerts sex- and context-dependent effects. In men, physiological androgen receptor signaling is generally hepatoprotective, whereas hypogonadism promotes insulin resistance, visceral adiposity and MASLD. Testosterone suppresses SREBP-1c-, FAS- and ACC-dependent lipogenesis; promotes peroxisome proliferator-activated receptor-α (PPARα), CPT1 and AMPK-dependent oxidation; and supports VLDL export through microsomal triglyceride transfer protein (MTTP) and apolipoprotein-B. It may also activate nuclear factor erythroid 2-related factor 2 (Nrf2), limit nuclear factor kappa-light-chain-enhancer of activated B cells (NF-κB)/TLR4 signaling and Kupffer-cell recruitment, and inhibit transforming growth factor-β (TGF-β)–SMAD-dependent stellate-cell activation [51].

Thus, physiological testosterone can restrain steatosis, inflammation, and fibrosis, whereas androgen deficiency and supraphysiological anabolic-androgenic steroid exposure may promote liver injury [51].

In women, the association is reversed: functional hyperandrogenism in PMOS promotes MASLD independently of body mass index by activating hepatic androgen receptor signaling, increasing fatty-acid synthesis, impairing insulin clearance, and accelerating steatosis [49].

### 3.5. Summary

MASLD progression reflects the convergence of hepatic lipid stress with systemic endocrine dysfunction (Table 1).

Disruption of hypothalamic–pituitary, pancreas–liver, thyroid–liver and gonad–liver signaling amplifies steatosis, inflammation and fibrosis, reinforcing the view of MASLD as a multisystem disorder. This endocrine framework supports mechanism-based interventions, including GH replacement in selected deficiency states, selective THR-β agonism and context-specific modulation of sex-hormone signaling. Integrating neuroendocrine phenotypes with liver staging may improve risk stratification and treatment selection. However, the current therapeutic landscape for hormone-based approaches in MASLD remains limited: resmetirom is the most firmly established example, whereas other endocrine-targeted strategies require further clinical evaluation.

## 4. Gut–Liver Axis

The gut–liver axis is a direct and consequential interorgan pathway in MASLD. Portal circulation continuously exposes the liver to intestinal nutrients, microbial products, bile acids and immunomodulatory metabolites [54]. Physiologically, this supports metabolic flexibility, immune tolerance and enterohepatic bile-acid recycling; barrier disruption and dysbiosis instead promote steatohepatitis, fibrosis and cirrhosis [55]. In metabolic dysfunction, diet-induced dysbiosis, barrier failure and altered microbial metabolism amplify steatosis, inflammation and fibrogenesis [54,56].

### 4.1. Disruption of the Intestinal Barrier: Microbial Danger Signals in MASLD

High-fat, high-fructose and ultra-processed dietary patterns can reduce microbial diversity, deplete barrier-supporting commensals and weaken tight junctions, increasing intestinal permeability. This metabolic endotoxemia facilitates portal translocation of pathogen-associated molecular patterns (PAMPs), including lipopolysaccharide (LPS), peptidoglycan and bacterial DNA [54,57]. Kupffer cells, hepatic stellate cells, sinusoidal endothelial cells and hepatocytes sense these signals through pattern-recognition receptors such as Toll-like receptor 4 (TLR4), Toll-like receptor 9 (TLR9) and nucleotide-binding oligomerization domain-like receptors [58]. Activation of Myeloid differentiation primary response 88 (MyD88)–NF-κB, interferon-regulatory and inflammasome pathways then promotes tumor necrosis factor (TNF), interleukin-1β (IL-1β), interleukin-6 (IL-6) and chemokines that recruit inflammatory monocytes and neutrophils, linking barrier failure to steatohepatitis and fibrotic progression [54,55].

### 4.2. From Dysbiosis to Hepatotoxic Signaling

Dysbiosis does not act solely by increasing microbial translocation; it also reshapes the metabolic output of the intestinal ecosystem [54,55]. MASLD and MASH have been associated with expansion of *Proteobacteria* and ethanol-producing taxa, relative depletion of butyrate-producing bacteria, and altered *Bacteroidetes*–*Firmicutes* relationships, although taxonomic signatures vary across geography, diet, medication exposure, and disease stage [55,59]. A recurring functional pattern is nevertheless emerging: loss of microbial functions that preserve mucosal integrity and expansion of pathways that generate hepatotoxic or pro-inflammatory metabolites [54]. Endogenous ethanol and acetaldehyde may aggravate oxidative stress and hepatocellular injury [60], whereas trimethylamine-derived trimethylamine-N-oxide (TMAO), branched-chain amino-acid (BCAA) derivatives, and imidazole propionate can impair insulin signaling and reinforce hepatic lipid accumulation [55,61]. Conversely, short-chain fatty acids (SCFAs), particularly butyrate and propionate, can support epithelial energy metabolism, regulatory T-cell differentiation, glucagon-like peptide-1 secretion, and AMPK activity, although their effects remain dose-, compartment-, and context-dependent [54,55].

### 4.3. Bile Acids Link Dysbiosis with Hepatic Injury

Bile acids provide a second major communication layer between gut microbes and hepatic metabolism. Primary bile acids synthesized in hepatocytes are modified by intestinal bacteria into secondary bile acids, which signal through nuclear and membrane receptors including farnesoid X receptor (FXR) and Takeda G protein-coupled receptor 5 (TGR5), thereby linking the liver, gut microbiota, and immune system [54,62]. In the healthy state, intestinal FXR–fibroblast growth factor 19 signaling restrains hepatic bile-acid synthesis, modulates gluconeogenesis and lipogenesis, and supports intestinal barrier integrity; recent experimental evidence further indicates that microbiota-dependent bile salt hydrolase activity can activate the intestinal FXR–fibroblast growth factor 19 (FGF19) axis and attenuate hepatic lipid accumulation [63]. In MASLD, microbial remodeling of the bile acid pool may blunt FXR activity, alter TGR5-dependent energy expenditure and immune signaling, and increase exposure to hydrophobic secondary bile acids such as deoxycholic acid [54,55]. These. These shifts can promote hepatocyte stress, stellate-cell activation, and a pro-fibrogenic niche, particularly when combined with lipotoxicity and innate immune activation. Conversely, ileal secondary bile-acid accumulation can activate TGR5–mTOR–oxidative phosphorylation signaling in CD8+ T cells, promote ileitis, impair enterohepatic circulation, and suppress hepatic FXR activity, thereby exacerbating MASLD progression [64].

### 4.4. Role of the Gut–Liver Axis Also in the Transition from Metabolic Stress to Fibrosis

Repeated exposure to LPS and other microbial ligands primes Kupffer cells and recruited macrophages, increasing transforming growth factor-β, platelet-derived growth factor, and reactive oxygen species; this portal delivery of PAMPs and microbial metabolites is increasingly viewed as a fibrogenic arm of the gut–liver axis [54,55]. These mediators activate hepatic stellate cells and portal fibroblasts, enhancing extracellular matrix deposition and tissue stiffness, consistent with evidence that gut-derived LPS and altered microbial metabolites can amplify hepatic inflammatory and fibrogenic cascades [54,65]. In parallel, dysregulated bile-acid signaling, microbial ethanol production, and reduced indole-mediated aryl hydrocarbon receptor activation may weaken epithelial repair mechanisms and perpetuate inflammatory exposure [60,64,66]. Thus, fibrosis in MASLD can be interpreted not only as the end product of hepatocellular lipotoxicity but also as the consequence of sustained portal delivery of microbial and metabolic danger signals [54,56].

### 4.5. Clinical Relevance

#### 4.5.1. MASLD Heterogeneity

Gut-derived signals may contribute to MASLD heterogeneity because microbial composition, barrier integrity and metabolite output vary among patients, influencing inflammation, fibrosis and treatment response [54,55]. Thus, similar hepatic fat burdens can accompany markedly different necroinflammatory and fibrotic trajectories, consistent with multi-omic evidence linking barrier dysfunction and host–microbiome interactions to MASLD severity in high-risk populations [57]. Microbiome composition, permeability, bile-acid profiles and markers of microbial exposure, including LPS-binding protein and soluble CD14, may complement liver-centered biomarkers. Integrated metagenomic, metabolomic, transcriptomic and fibrosis data are beginning to define microbial–host endotypes, although human causality remains confounded by diet, drugs, obesity, diabetes and genetics [56,67,68]. No validated clinical classification based on crosstalk endotypes is yet available.

#### 4.5.2. Therapeutics

Fiber-rich, minimally processed plant-based diets can enrich short-chain fatty-acid producers, reduce endotoxemia and improve insulin sensitivity [54,55]. Probiotics, synbiotics and postbiotics may improve aminotransferases, steatosis indices and inflammation, but optimal strains, duration and patient selection remain uncertain [69,70]. Fecal microbiota transplantation demonstrates ecosystem remodeling, although donor variability, durability, safety and inconsistent trial results limit its use in MASLD [71,72]. Pharmacological targeting of bile-acid signaling, intestinal FXR, incretin pathways and microbial metabolites may offer more controllable, endotype-guided strategies [54,63,73].

#### 4.5.3. Summary

**Figure 1:**
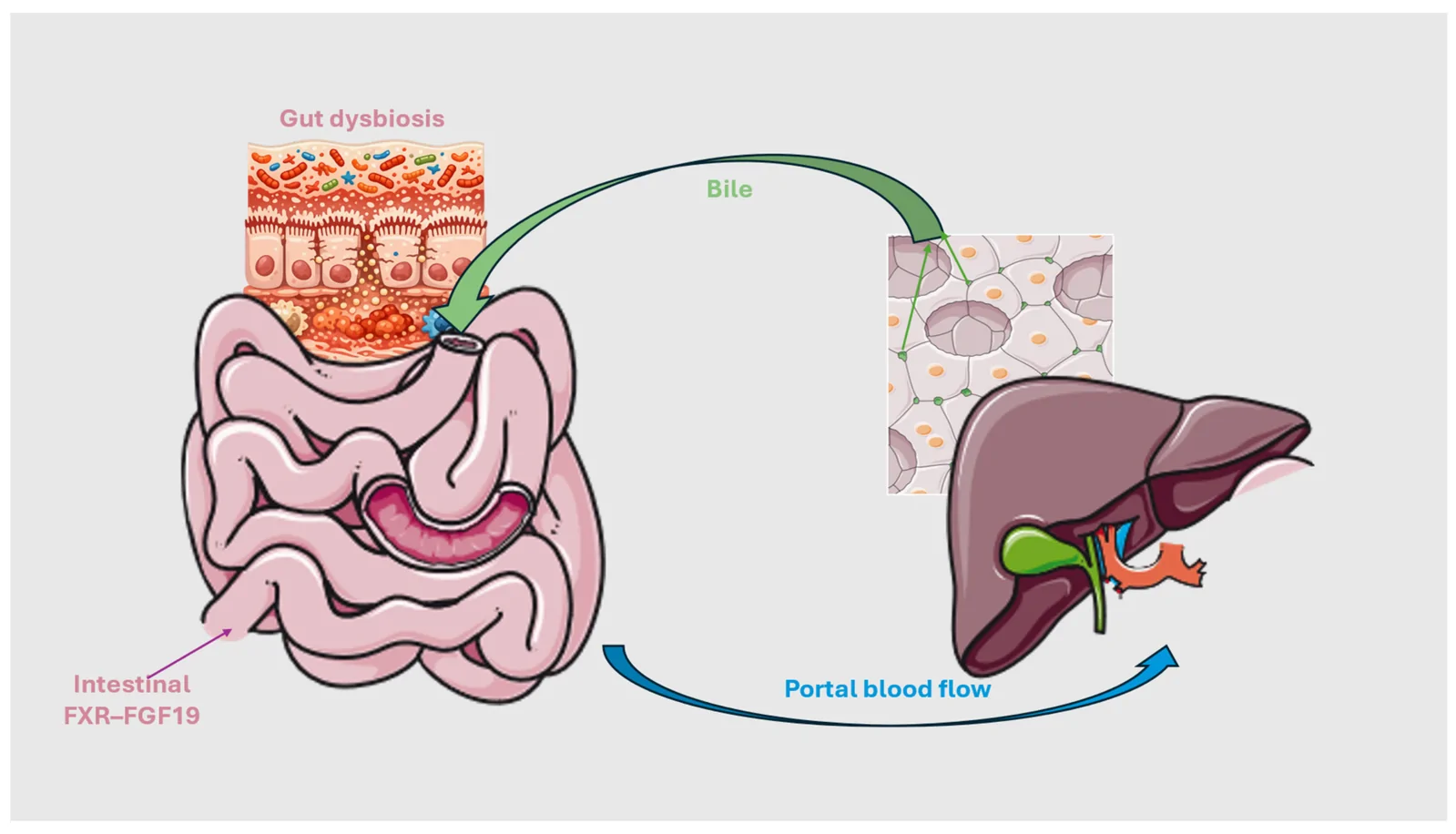
Bidirectional gut–liver signaling in MASLD. Intestinal dysbiosis and impaired epithelial barrier integrity promote portal translocation of lipopolysaccharide and other microbial products/metabolites, contributing to MASLD. Conversely, liver-derived bile acids signal through intestinal farnesoid X receptor (FXR), inducing fibroblast growth factor 19 (FGF19) and providing endocrine feedback to the liver. Original image drawn with Microsoft 365 Copilot (last updated on 4 August 2026) and modified with Servier Medical Art (https://smart.servier.com/), licensed under CC BY 4.0 (https://creativecommons.org/licenses/by/4.0/, accessed on 24 August 2026).

The gut–liver axis reframes MASLD as an ecological network integrating host metabolism, diet, microbes and mucosal immunity. The intestine supplies both injurious and protective signals, while the liver shapes the gut through bile acids, immune mediators and metabolic regulation. Defining this reciprocal circuitry is essential to identify causal, stage-specific targets. Gut-directed strategies will probably complement, rather than replace, liver-directed and cardiometabolic therapies by reducing inflammation, limiting fibrosis and personalizing care. Figure 1 illustrates the pathophysiology of the gut–liver axis.

## 5. Adipose Tissue–Liver Axis

### 5.1. Adipose Failure and Hepatic Lipid Overflow

The adipose tissue–liver axis is a dominant metabolic circuit in MASLD because white adipose tissue determines the magnitude, timing, and inflammatory context of lipid delivery to the liver [73,74]. In healthy energy surplus, subcutaneous adipose tissue buffers excess nutrients through adipocyte hyperplasia, triglyceride storage, and insulin-sensitive suppression of lipolysis. When this storage compartment becomes hypertrophic, hypoxic, and inflamed, its buffering capacity fails, and non-esterified fatty acids are released chronically into the circulation. Adipose-derived fatty acids are a major source of hepatic triglyceride stores in MASLD [75]. The liver is then exposed to a sustained substrate flux that exceeds mitochondrial oxidative capacity and very-low-density lipoprotein (VLDL) export, favoring hepatocellular triglyceride accumulation, toxic lipid intermediates, oxidative stress, and endoplasmic-reticulum stress [17,73]. This substrate-driven view has been reinforced by recent high-impact syntheses that position white adipose-tissue insulin resistance, non-esterified fatty-acid flux, gut-derived metabolites, and hepatic mitochondrial dysfunction as integrated determinants of steatosis, MASH progression, and treatment heterogeneity [73,76].

### 5.2. Adipose Insulin Resistance Drives Steatogenesis

In insulin-sensitive adipocytes, postprandial insulin restrains hormone-sensitive lipase and adipose triglyceride lipase, thereby limiting release of fatty acids during feeding; impaired suppression of adipose lipolysis is a hallmark of MASLD and correlates with steatosis, necroinflammation, and fibrosis [75]. In obesity-associated adipose dysfunction, this antilipolytic action is blunted, creating a paradoxical state in which hyperinsulinemia coexists with persistent lipolysis and increased non-esterified fatty-acid flux to the liver [73,74]. The liver therefore receives both an excess of fatty acids and an insulin signal that remains sufficiently active to induce SREBP-1c and ChREBP response element-binding protein, amplifying DNL [73,77]. This dual input, increased adipose-derived lipid delivery and preserved hepatic lipogenic signaling, helps explain why MASLD can progress despite systemic insulin resistance. Visceral adipose tissue is particularly relevant because its venous drainage and inflammatory phenotype promote direct metabolic coupling with the liver, whereas limited expandability of subcutaneous adipose tissue favors ectopic lipid deposition across liver, muscle, and pancreas [17,76].

### 5.3. Adipose Tissue as an Immune–Endocrine Organ

Expansion of hypertrophic adipocytes is accompanied by mechanical stress, impaired vascularization, local hypoxia, and cell death, which recruit monocyte-derived macrophages and promote crown-like structures around dying adipocytes [78]. These macrophage-rich niches release tumor necrosis factor, interleukin-1β, interleukin-6, and chemokines that propagate systemic low-grade inflammation and impair insulin receptor signaling in adipose tissue and liver [79]. In parallel, adipocytes and immune cells alter the adipokine milieu. Adiponectin, which normally activates AMP-activated protein kinase and peroxisome proliferator-activated receptor-α pathways, enhances fatty-acid oxidation and restrains stellate-cell activation, is reduced in obesity and advanced MASLD [80,81]. Conversely, leptin, resistin, chemerin and other pro-inflammatory adipokines may promote hepatic inflammation, endothelial dysfunction and fibrogenic signaling, although their effects depend on disease stage, receptor expression and metabolic context [80]. The clinical importance of this inflammatory adipose–liver interface is underscored by recent synthesis of MASLD as a multisystem metabolic disease [17].

### 5.4. Mediators of the Adipose Tissue–Liver Axis

Beyond cytokines and adipokines, adipose tissue signals to the liver through extracellular vesicles, bioactive lipids and microRNAs. Adipocyte-derived extracellular vesicles transfer regulatory proteins, lipids and RNAs to distant metabolic tissues, influencing insulin sensitivity and systemic homeostasis [82]. Insulin regulates the sorting and release of adipocyte small-extracellular-vesicle microRNAs linked to obesity and insulin resistance, suggesting that hyperinsulinemia remodels adipose–liver communication [83]. In MASLD, adipocyte small-extracellular-vesicle-derived microRNA-30a-3p worsens hepatocyte lipotoxicity and steatosis, whereas adipose-tissue macrophage-derived small extracellular vesicles enriched in miR-155 and miR-34a activate hepatic stellate cells and promote fibrosis [84,85]. Thus, this axis transmits both fuels and information that reprogram hepatocyte metabolism, macrophage polarization and extracellular matrix remodeling. The steatotic liver reciprocally signals to extrahepatic organs through hepatokines, dyslipidemia, glucose overproduction, procoagulant factors and circulating microRNAs [76,86].

### 5.5. Role in Hepatic Fibrogenesis

Saturated fatty acids, ceramides and diacylglycerols induce mitochondrial overload, reactive oxygen species production and hepatocyte ballooning; dying hepatocytes release damage-associated molecular patterns that activate Kupffer cells and recruit inflammatory monocytes [17,73]. In this primed hepatic environment, adipose-derived cytokines and leptin can enhance transforming growth factor-β and platelet-derived growth factor signaling, promoting hepatic stellate-cell activation and matrix deposition [87]. In contrast, adiponectin deficiency removes an antifibrotic brake by reducing AMPK-dependent lipid oxidation and weakening inhibition of stellate-cell proliferation [88]. Thus, the adipose tissue–liver axis links the earliest metabolic lesion of excess lipid storage to the later architectural lesion of fibrosis. Importantly, this relationship is bidirectional: as hepatic inflammation progresses, the liver contributes to systemic insulin resistance through altered hepatokine secretion, impaired lipid handling and increased inflammatory mediators, thereby worsening adipose dysfunction and closing a self-reinforcing loop [73,89].

### 5.6. Clinical Relevance

#### 5.6.1. MASLD Heterogeneity

This axis helps explain MASLD heterogeneity in progression, cardiometabolic associations and treatment response [76,90]. Despite similar body mass index, patients differ in adipose distribution, expandability, insulin sensitivity, immune composition and adipokine profiles, making adipose function more informative than body size alone [74,76]. Visceral adiposity is more strongly associated with cardiometabolic risk, hepatic fat and fibrosis, whereas healthier subcutaneous expansion may limit ectopic lipid deposition [10,90]. These findings support adipose-function-centered rather than weight-centered phenotyping. Adiponectin, the leptin-to-adiponectin ratio, inflammatory mediators, lipidomic signatures and imaging of visceral and subcutaneous adiposity may improve risk stratification when combined with liver stiffness, genetic variants and cardiometabolic comorbidities [10,76].

#### 5.6.2. Therapeutics

Sustained weight loss remains central to adipose tissue–directed MASLD therapy because it improves adipose insulin sensitivity, lowers free-fatty-acid flux, restores adipokine balance and reduces hepatic steatosis; greater loss is generally needed for steatohepatitis resolution and fibrosis regression [10,90]. Exercise improves adipose inflammation, insulin responsiveness and cardiometabolic risk even with modest weight loss, supporting lifestyle intervention as a mechanism-based therapy [10,76]. Incretin-based therapies reduce adipose mass, improve glycemic control and lower hepatic fat; in MASH with stage F2–F3 fibrosis, semaglutide and tirzepatide have shown histological benefits consistent with systemic metabolic remodeling [90,91,92]. Bariatric surgery provides the strongest evidence that durable correction of adipose dysfunction improves MASLD, although careful selection, perioperative assessment and long-term nutritional monitoring remain essential [10,90]. Future strategies may move beyond weight loss to restore adipose health by enhancing expandability, reducing macrophage activation, modulating adipokines and interrupting vesicle-mediated inflammation [76].

#### 5.6.3. Summary

Conceptually, adipose tissue should therefore be viewed not as a passive fat reservoir but as an active determinant of hepatic destiny. Its capacity to safely store energy, restrain lipolysis, maintain an anti-inflammatory adipokine profile, and communicate appropriately with immune and metabolic networks determines whether nutrient excess remains metabolically contained or is exported to the liver as lipotoxic and inflammatory stress. Dissecting the molecular grammar of adipose–liver communication will be essential for precision hepatology, because patients with adipose-driven MASLD may require therapies that restore adipose tissue function as much as agents that directly target hepatic steatosis, inflammation, or fibrosis.

## 6. Liver–Brain Axis

### 6.1. Definitions, Mechanisms and Outcomes

Communication across the liver–brain axis is mediated by overlapping neural, endocrine, metabolic, immune and microbial routes. Afferent and efferent vagal fibers provide a rapid anatomical conduit by which hepatic sensory information reaches central autonomic nuclei and brain-derived outputs subsequently modulate hepatic glucose production, lipid handling and inflammatory tone [93,94,95,96]. In parallel, circulating nutrients, lipids and hepatokines convey the metabolic state of the liver to the brain [95,97]. When hepatic detoxification and immunometabolic control are impaired, ammonia, microbial products and systemic inflammatory mediators can evade hepatic clearance, enter the systemic circulation and disturb blood–brain barrier integrity, microglial activation and neuronal function [98]. This circuitry is embedded within the broader gut–liver–brain axis, in which intestinal dysbiosis, bile acids and microbial metabolites modify hepatic inflammation and, secondarily, brain health [99].

Disruption of this axis produces a spectrum of neurological consequences. At one extreme, decompensated cirrhosis and portosystemic shunting give rise to the canonical syndrome of hepatic encephalopathy, driven by impaired ammonia handling, systemic inflammation, blood–brain barrier dysfunction and glial activation [98,100,101]. In MASLD, however, brain involvement is likely to emerge earlier and more insidiously, through chronic low-grade inflammation, insulin resistance, dyslipidemia, altered bile-acid and amino-acid metabolism, endothelial dysfunction, impaired ketogenesis and disrupted neurotrophic signaling. Together, these pathways provide a biological rationale for fatigue, sleep disturbance, impaired attention, mood symptoms, cognitive decline and brain atrophy before advanced liver failure becomes clinically evident [96,102].

MASLD therefore extends the liver–brain paradigm beyond the setting of advanced liver failure. It highlights how hepatic metabolic stress and inflammatory activation can influence neural homeostasis during earlier disease stages. Hepatic inflammation may propagate neuroinflammatory responses associated with cognitive dysfunction, behavioral alterations and brain atrophy, whereas reduced brain-derived neurotrophic factor signaling may weaken adaptive liver–brain communication and compromise neural resilience [102,103,104,105].

### 6.2. Relevance for MASLD Heterogeneity

The liver–brain framework extends MASLD heterogeneity beyond steatosis, inflammation and fibrosis to neurovascular, neuroimmune and neuroendocrine susceptibility. Despite similar hepatic fat, patients may differ in fatigue, sleep disruption, cognition and dementia vulnerability because liver-derived signals vary with inflammation, fibrosis, diabetes, vascular disease, sarcopenia, endothelial dysfunction and systemic metabolic load [102]. Brain-derived autonomic and endocrine outputs reciprocally regulate appetite, energy expenditure, sympathetic tone, adipose lipolysis, cortisol and insulin, thereby modifying hepatic lipid handling and inflammation. Sex and endocrine context, including menopause, androgen deficiency, PMOS, hypothalamic–pituitary disruption and long-standing T2D, further shape this circuitry [96].

Neurocognitive manifestations therefore reflect interacting metabolic, vascular and inflammatory networks rather than hepatic fat alone. Compromised brain health in MASLD probably arises from convergent systemic inflammation, vascular disease, brain ageing and neurodegeneration [102], with the liver communicating with the central nervous system through gut–liver–brain pathways, immune mediators, metabolites and neural circuits [96].

Meta-analytic and population-based evidence supports this model. A systematic review and meta-analysis of seven studies and 891,562 individuals linked NAFLD to cognitive impairment, but not significantly to all-cause dementia or Alzheimer disease, suggesting that subtle dysfunction may precede diagnosed dementia [106]. Another meta-analysis of 2,345,929 participants found a modest association between NAFLD and incident dementia, with substantial study heterogeneity [107]. Prospective analyses using MAFLD and MASLD definitions further associate MASLD mainly with vascular dementia, particularly in diabetes-defined and lean metabolic subtypes, whereas links to Alzheimer disease are weaker or absent [108].

Dementia risk appears to track chronic liver disease severity rather than steatosis alone. In a meta-analysis of eight longitudinal cohorts comprising 1,115,759 middle-aged individuals, including 31,129 with baseline liver fibrosis, fibrosis predicted a 32% higher long-term dementia risk over a median 14 years, independent of demographic, social, anthropometric and cardiometabolic confounders; risk increased with fibrosis severity [109].

Neurological outcomes in MASLD thus reflect individual variation in vascular, inflammatory, glycemic and neuroendocrine pathways, explaining why some patients develop fatigue, cognitive slowing or vascular brain injury while others remain resilient despite similar steatosis.

### 6.3. Impact on Disease Stratification and Therapeutics

Because dementia is multifactorial and disease-modifying therapies remain limited, prevention depends on actionable risk markers. Liver fibrosis may indicate systemic vulnerability [110], identifying patients at moderately increased cognitive risk who could benefit from closer surveillance and risk-factor modification. In MASLD, the liver–brain axis extends stratification to neurocognitive vulnerability, autonomic tone, sleep, mood and vascular brain risk, although this framework remains hypothesis-generating. Cognitive dysfunction probably reflects convergent systemic inflammation, insulin resistance, endothelial dysfunction, altered bile-acid and amino-acid metabolism, impaired ketogenesis, gut–liver–brain dysbiosis and neurodegenerative susceptibility rather than hepatic fat alone [96,102]. Clinically, fibrosis, diabetes, vascular risk, sarcopenia, sleep quality and cognition should be assessed alongside liver biomarkers, especially in high-risk patients with disproportionate fatigue, sleep or cognitive symptoms. Treatment should integrate liver-directed therapy with cardiometabolic, vascular, behavioral, endocrine and gut-directed strategies, including weight loss, physical activity, optimized diabetes control, incretin-based therapies, selective thyroid hormone receptor-β agonism, correction of endocrine drivers, microbiome modulation and vascular-risk prevention. Trials should test whether improved MASLD histology also normalizes liver–brain signaling and improves cognition, fatigue, sleep, quality of life and long-term neurocognitive risk.

## 7. Liver–Kidney Axis in MASLD

### 7.1. Definitions and Burden

MASLD encompasses a continuum from simple steatosis to MASH, progressive fibrosis, cirrhosis and HCC [10]. CKD, conversely, is defined by persistent structural or functional kidney abnormalities, most commonly reduced estimated glomerular filtration rate and/or albuminuria, and is staged functionally rather than histologically [111]. The liver–kidney axis in MASLD therefore refers not merely to the late hepatorenal syndrome of decompensated cirrhosis, but to a broad, bidirectional and often subclinical network linking steatotic, inflamed or fibrotic liver tissue with glomerular, tubular, vascular and interstitial renal injury. This physiological axis often remains clinically invisible until portal hypertension becomes manifest as ascites, refractory ascites or hepatorenal syndrome, but non-cirrhotic NAFLD/MASLD has been associated since 2008 with higher CKD risk independent of obesity, T2D and other conventional renal-risk factors [111]. Its epidemiological relevance is considerable: MASLD affects roughly one third of adults worldwide, while CKD affects approximately 10% of the global population and accounts for substantial mortality and years of life lost; in high-income settings CKD is most often linked to metabolic disorders such as T2D, obesity and hypertension, whereas infectious and environmental causes contribute more prominently in lower-income settings [111].

### 7.2. Epidemiological Evidence

Epidemiological evidence consistently links MASLD with a higher burden of CKD, particularly in the presence of MASH or advanced fibrosis [110,112,113]. Although this association partly reflects shared cardiometabolic risk factors—including obesity, T2D, hypertension, dyslipidemia and ageing—longitudinal studies and meta-analyses indicate that MASLD is associated with incident CKD independently of these conventional determinants [113] In an updated meta-analysis of 13 observational studies, comprising 1,222,032 participants and 33,840 incident CKD cases, NAFLD was associated with an approximately 45% higher long-term risk of incident CKD stage ≥ 3 after adjustment for age, sex, obesity, hypertension, diabetes and other established renal-risk factors [114]. Thus, CKD should be viewed not simply as a comorbidity of MASLD, but as part of its systemic cardiometabolic phenotype. This concept is reinforced by cardiovascular-outcome data: patients with coexistent MASLD and CKD carry an especially adverse risk profile, characterized by greater obesity, hypertension, dyslipidemia and diabetes burden, and by increased risk of coronary heart disease and heart failure [115]. Liver fat severity, renal function, sex and menopausal status may also interact to shape subclinical atherosclerosis, suggesting that the liver–kidney–metabolic axis is modified by biological sex and reproductive ageing [116]. A meta-analysis by Kueh and colleagues further quantifies this overlap: across 36 observational studies, CKD affected 15.22% of adults with MASLD, with an incidence of 22.17 cases per 1000 person-years; diabetes, hypertension and dyslipidemia substantially increased CKD odds, and coexistence of MASLD and CKD more than doubled all-cause mortality compared to MASLD alone [117]. Collectively, these data position CKD as a frequent, prognostically meaningful component of the liver–kidney–cardiometabolic axis, rather than an incidental extrahepatic finding.

### 7.3. Mechanisms

The liver–kidney axis in MASLD reflects shared cardiometabolic injury and liver-derived systemic signaling. Insulin resistance, adipose dysfunction, chronic inflammation, oxidative and endoplasmic-reticulum stress, mitochondrial dysfunction and renin–angiotensin–aldosterone system activation promote both steatohepatitis and renal microvascular, glomerular and tubulointerstitial injury [112,118]. The steatotic and fibrotic liver can further damage the kidney through hepatokines, cytokines, procoagulant mediators, extracellular vesicles, dyslipidemia and altered hemodynamics; progressive fibrosis and subclinical portal hypertension also promote neurohumoral activation, renal vasoconstriction, sodium retention and reduced renal functional reserve [111,119]. Intestinal dysbiosis, increased permeability, lipopolysaccharide translocation and microbial metabolites such as indoxyl sulfate, p-cresyl sulfate and trimethylamine-N-oxide further amplify systemic inflammation, endothelial dysfunction, bile-acid dysregulation and renal fibrogenesis [120]. Accordingly, liver fibrosis, rather than steatosis alone, aligns most closely with CKD risk, cardiovascular amplification and adverse systemic outcomes [114,118].

Renal injury reciprocally worsens hepatic oxidative stress, inflammation, endothelial dysfunction, uremic-toxin accumulation, lipid handling and gut microbial metabolism. Potential renal phenotypes include diabetic nephropathy and obesity-related glomerulopathy, but renal histology studies in MASLD remain scarce, so research relies mainly on eGFR and proteinuria [111]. Even before cirrhosis, subclinical portal hypertension may impair renal vasoregulation through a hepatorenal reflex, sympathetic activation, renal vasoconstriction, sodium retention and reduced renal functional reserve [111]. These pathways converge with renin–angiotensin–aldosterone system activation, oxidative and endoplasmic-reticulum stress, mitochondrial dysfunction, inflammasome signaling, adipose-tissue dysfunction and transforming growth factor-β pathways shared by MASH, diabetic kidney disease and obesity-related glomerulopathy.

### 7.4. Relevance for MASLD Heterogeneity

This framework helps explain MASLD heterogeneity. Despite similar hepatic fat, trajectories vary with renal reserve, albuminuria, fibrosis stage, adipose distribution, diabetes duration, genetics, diet, microbiome, sex-hormone and menopausal status, sarcopenia, alcohol exposure and socioeconomic context [76]. Conversely, occult advanced MASLD or MASH-related fibrosis in CKD may identify patients at disproportionate cardiovascular risk. Thus, the liver–kidney axis is both a disease-amplifying circuit and a stratification framework for clustered hepatic, renal and cardiometabolic risk that may be underestimated by conventional cardiovascular scores.

### 7.5. Implications for Treatment

Lifestyle measures remain foundational, but treatment should be integrated rather than organ-specific. Statins should be used when indicated; renin–angiotensin–aldosterone system inhibitors are central for albuminuric CKD and hypertension; and sodium–glucose cotransporter-2 inhibitors reduce kidney and heart-failure outcomes and may indirectly improve hepatic steatosis [121,122]. Glucagon-like peptide-1 receptor agonists and dual incretin agonists promote weight loss, improve glycemia and benefit steatohepatitis and cardiometabolic risk, with semaglutide achieving greater NASH resolution than placebo [123]. Emerging MASH therapies, including resmetirom for non-cirrhotic MASH with significant fibrosis, should incorporate renal and cardiovascular outcomes; MAESTRO-NASH showed superior NASH resolution and fibrosis improvement versus placebo [10,124]. Microbiome- and bile-acid-targeted therapies, fiber enrichment, non-lethal choline analogues, FXR agonists and precision nutrition remain promising but require cross-organ trials [120,125]. Clinically, MASLD care should include routine eGFR and albuminuria assessment, while CKD and diabetes clinics should assess liver fibrosis. Future trials should evaluate MASLD, CKD and CVD as integrated outcomes.

### 7.6. Summary

**Figure 2:**
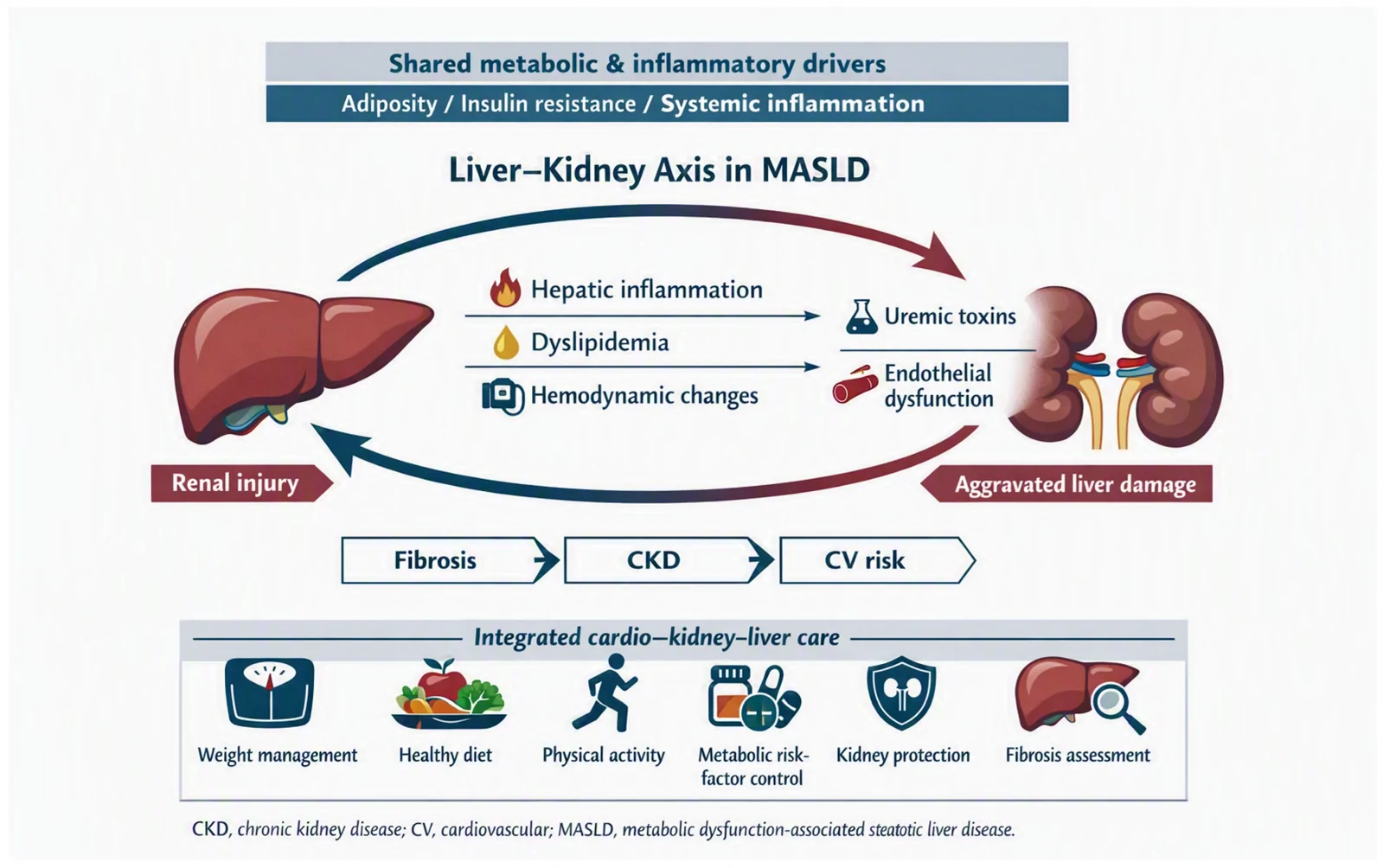
Liver–kidney axis in MASLD. Shared metabolic and inflammatory drivers promote bidirectional crosstalk between the liver and kidneys. Hepatic inflammation, dyslipidemia, and hemodynamic changes contribute to renal injury, whereas uremic toxins and endothelial dysfunction may aggravate liver damage. This interaction promotes fibrosis, CKD, and CV risk, supporting integrated cardio–kidney–liver care based on weight management, healthy diet, physical activity, metabolic risk-factor control, kidney protection, and fibrosis assessment. Abbreviations: CKD, chronic kidney disease; CV, cardiovascular; MASLD, metabolic dysfunction-associated steatotic liver disease.

To sum up, the liver–kidney axis reframes MASLD as a systemic cardio–renal–metabolic disorder rather than a liver-confined disease. Hepatic steatosis, inflammation and fibrosis interact with adipose dysfunction, insulin resistance, gut-derived metabolites, endothelial injury and neurohumoral activation to promote renal vulnerability, whereas CKD feeds back on the liver through uremic toxins, oxidative stress, vascular inflammation and cardiometabolic instability. This bidirectional circuitry helps explain heterogeneity in MASLD progression and supports integrated risk stratification using fibrosis stage, albuminuria, estimated glomerular filtration rate, diabetes control and vascular risk. Clinically, the axis argues for combined cardio–kidney–liver–metabolic care and for trials that evaluate whether targeting one node of this network can improve outcomes across organs. Figure 2 schematically illustrates the pathophysiology interconnection of the liver–kidney axis.

## 8. MASLD–Muscle–Bone Axis

### 8.1. Definitions

The MASLD–muscle–bone axis links steatotic and inflamed liver tissue with muscle quantity and quality, bone mass and turnover, and fracture risk. Sarcopenia denotes reduced muscle strength with low muscle quantity or quality and, when severe, impaired physical performance [126]. Myosteatosis is ectopic lipid infiltration within and between muscle fibers and may occur despite preserved muscle mass [127]. Osteopenia and osteoporosis denote reduced bone mineral density, whereas osteosarcopenia combines low bone mass with sarcopenia [128]. In MASLD, these conditions reflect systemic insulin resistance, inflammation, endocrine dysregulation and ectopic lipid deposition, and are associated with liver activity, fibrosis and cardiometabolic risk [127,129,130].

Muscle mass and quality are distinct. Sarcopenia is assessed using appendicular skeletal-muscle mass, grip strength, chair-stand performance or gait speed; myosteatosis is evaluated by computed tomography attenuation, MRI proton density fat fraction, ultrasound echogenicity or, less often, biopsy and lipid extraction [131].

Bone health is assessed by dual-energy X-ray absorptiometry, fracture history and turnover markers, but higher body weight may preserve or increase areal bone mineral density despite declining bone quality and fracture resistance [132]. Precision assessment should therefore integrate muscle strength and fat infiltration, bone quantity and quality, and liver fibrosis rather than rely on body mass index or liver fat alone [127,128,133].

### 8.2. Epidemiology

Sarcopenia commonly coexists with MASLD, especially in older adults and those with T2D, obesity, metabolic syndrome or advanced fibrosis. The relationship is reciprocal: metabolic syndrome promotes both conditions, whereas muscle loss worsens insulin resistance, reduces glucose disposal and increases hepatic lipid availability [129]. Muscle loss also predicts adverse outcomes. Among 19,867 adults with MASLD but no baseline CKD, sarcopenia predicted incident CKD, with progressive loss conferring dose-dependent risk, particularly with severe fibrosis [134]. Thus, muscle depletion is integral to the broader cardio–kidney–liver–metabolic phenotype.

Myosteatosis may be more informative than muscle mass. It is detectable in non-cirrhotic MASLD and correlates with disease activity when assessed by computed tomography [127]. Obese individuals may retain muscle mass despite poor quality, reduced function and impaired insulin-stimulated glucose uptake, whereas lean individuals may have an adverse fat-to-muscle phenotype missed by body mass index. The fat-to-muscle ratio may therefore improve risk prediction by capturing ectopic adiposity relative to muscle reserve [135].

Bone involvement is similarly heterogeneous. Meta-analyses associate NAFLD/MASLD with osteoporosis and fractures, although bone mineral density varies by sex, site, ethnicity and adiposity. One found lower bone mineral density and greater osteoporosis and fracture risk, particularly at the femoral neck and total hip in men, with ethnic differences [133]. A later synthesis reported higher femur bone mineral density in some subgroups, especially women and overweight individuals, yet greater osteoporosis or fracture risk and lower turnover markers [132]. Thus, bone mineral density may underestimate fragility in metabolically inflamed, low-turnover bone.

Sex strongly modifies this epidemiology. Men have higher MASLD prevalence before midlife and may be more vulnerable to adverse muscle–liver coupling as muscle mass declines; Japanese data linked low skeletal-muscle mass index and elevated phase angle to MASLD in men but not women [136]. After menopause, loss of estrogenic protection promotes visceral adiposity, insulin resistance, bone loss, and potentially more severe MASLD. Regional composition also matters: leg fat-to-muscle ratio may better indicate MASLD risk in women, whereas other patterns may better predict liver cancer or liver-related outcomes in men [135]. The MASLD–muscle–bone axis is therefore sexually dimorphic and cannot be defined by a universal muscle-mass threshold.

### 8.3. Mechanisms

The MASLD–muscle–bone axis is sustained by a self-reinforcing cycle of ectopic lipid deposition, insulin resistance and inflammation. Muscle loss or myosteatosis reduces insulin-stimulated glucose disposal and mitochondrial oxidation, increasing glucose, insulin and substrate flux to hepatic de novo lipogenesis. Hepatic steatosis and MASH then amplify systemic inflammation, dyslipidemia and hepatokine release. Thus, myosteatosis both marks and promotes hepatic lipid overload, muscle dysfunction and inactivity [127,137].

Crosstalk is mediated by organ-derived proteins and vesicular signals, but the muscle-specific balance is especially important. Altered myostatin, irisin, interleukin-6, interleukin-15, apelin and brain-derived neurotrophic factor modify insulin sensitivity, lipid oxidation, mitochondrial biogenesis and inflammation [138,139,140]. Exercise-induced myokines generally support hepatic lipid oxidation, whereas myostatin, tumor necrosis factor-α and chronic interleukin-6 signaling impair muscle anabolism and promote sarcopenia and hepatic fibrogenesis [141].

Hepatic FGF21 is often elevated in MASLD, indicating metabolic stress and possible FGF21 resistance; high levels may therefore mark severity without restoring lipid oxidation or muscle health [142].

Bone actively contributes through osteocalcin, osteopontin, sclerostin and FGF23, which link remodeling to glucose metabolism, inflammation, phosphate homeostasis and liver injury. Low osteocalcin may impair insulin secretion and sensitivity, whereas elevated osteopontin and FGF23 associate with inflammatory and fibrogenic phenotypes. Inflammation, oxidative stress, vitamin D deficiency, altered sex steroids, growth hormone–IGF-1 disruption, reduced loading and gut dysbiosis promote bone loss, fragility and osteosarcopenia [143,144]. Sarcopenia and osteopenia therefore reinforce each other: muscle loss reduces anabolic loading, increases falls and removes bone-supportive myokine signals [130].

Sex-hormone effects described in Section 3 also modify muscle and bone: loss of estrogen after menopause promotes visceral and ectopic fat, skeletal imbalance and a combined MASLD–osteopenia–sarcopenia phenotype, whereas hypogonadism in men promotes visceral adiposity, insulin resistance, sarcopenia and low bone mass [51,130,145,146,147]. These differences support sex- and reproductive-stage-specific thresholds and treatment priorities.

Gut-derived inflammation and metabolites further modify this axis, linking dysbiosis to impaired muscle insulin signaling, mitochondrial function and bone remodeling [148,149,150].

Physical inactivity and poor diet amplify this loop, whereas fiber-rich diets and exercise may restore microbial diversity, improve muscle metabolism and reduce hepatic fat. Low protein or energy intake and calcium or vitamin D deficiency can coexist with obesity and promote sarcopenia and osteopenia [151,152,153]. In a small MASLD cohort, sarcopenia was common and associated with lower calcium intake, underscoring the importance of diet quality beyond caloric excess [154].

### 8.4. Clinical Implications for Precision-Medicine Approaches

Recognition of the MASLD–muscle–bone axis broadens clinical phenotyping beyond liver enzymes, steatosis and fibrosis to muscle strength and quality, physical performance, bone density, fracture risk, nutrition, sex hormones and menopausal status [129,130]. This is especially relevant in older adults, lean MASLD, postmenopausal women, men with hypogonadism, patients with T2D, those undergoing rapid pharmacological or surgical weight loss and those with advanced fibrosis, in whom weight loss may reduce hepatic fat but worsen sarcopenia, frailty and fracture risk.

Precision assessment should combine non-invasive liver fibrosis tests with body composition and function. Opportunistic computed tomography or MRI can quantify muscle area and attenuation; DXA assesses appendicular lean mass and bone mineral density; bioelectrical impedance estimates skeletal-muscle mass and phase angle; grip strength and chair-stand tests assess function; and fracture-risk algorithms add skeletal risk [155,156,157]. Fat-to-muscle ratio and regional muscle adiposity require standardized cut-offs and sex- and ethnicity-specific validation before routine use.

This framework also refines prognosis. Myosteatosis and sarcopenia identify systemic metabolic failure beyond isolated steatosis [127,129], whereas osteopenia or osteosarcopenia indicate risks of falls, fractures, disability and poor tolerance of intensive weight loss [128,158]. Because muscle mass, bone density and liver fat have sex- and life-stage-specific implications [159,160], precision hepatology should integrate liver fibrosis, cardiometabolic burden, muscle reserve and skeletal fragility.

### 8.5. Principles of Treatment

Treatment should target the axis rather than one organ. Structured exercise is the central intervention because it simultaneously reduces hepatic fat, improves insulin sensitivity, increases or preserves muscle strength, reduces myosteatosis and supports bone loading [129,161]. Aerobic exercise improves hepatic lipid oxidation and cardiorespiratory fitness; resistance training is essential for muscle protein synthesis, strength and bone health; combined programs are likely to be most effective, particularly when individualized for age, frailty, sex, comorbidities and baseline physical performance [161,162]. Mechanistically, exercise activates AMPK, PGC-1α, Nrf2 and Akt pathways, enhances mitochondrial biogenesis, reduces oxidative stress and remodels myokine signaling toward an insulin-sensitizing and anti-inflammatory profile [139].

Nutrition should combine liver-directed weight reduction with musculoskeletal preservation. Energy restriction can reduce hepatic and muscle fat, but it should be accompanied by adequate protein intake, resistance exercise, and correction of vitamin D and calcium deficiency to avoid lean-mass loss and skeletal fragility [158,163]. Mediterranean-style dietary patterns, minimally processed foods, fiber enrichment, and adequate high-quality protein are preferable to purely calorie-centered prescriptions [152,153].

In older adults and postmenopausal women, aggressive weight loss should be balanced against the risk of sarcopenia and bone loss; in men with hypogonadism or low muscle reserve, endocrine assessment might be relevant [147,164]. Bariatric surgery and incretin-based pharmacotherapy can markedly improve MASLD, but both require planned monitoring of muscle mass, strength, nutritional status and bone health [165,166].

Pharmacological strategies remain indirect but increasingly relevant. Incretin-based therapies, thyroid hormone receptor-β agonists, insulin sensitizers and lipid-lowering therapies might improve the hepatic component of the axis, whereas osteoporosis therapies should be used according to standard fracture-risk indications [130,167,168]. Future agents that target myostatin signaling, mitochondrial dysfunction, FGF21 pathways, adiponectin signaling or gut-derived inflammatory cues might modify both MASLD and musculoskeletal decline, but clinical trials should include endpoints for muscle quality, physical function and bone fragility rather than liver histology alone [73,129,169,170]. Ultimately, remission of MASLD should not be defined only by reduced hepatic fat or fibrosis improvement, but by restoration of metabolic resilience across liver, muscle and bone.

### 8.6. Summary

The MASLD–muscle–bone axis provides a unifying framework for understanding why patients with similar liver fat content can have markedly different trajectories. Myosteatosis captures the quality of skeletal muscle, sarcopenia captures the loss of muscle reserve, and osteopenia or osteosarcopenia captures skeletal fragility within the same systemic disease network. Sex, ageing and reproductive endocrine status modify every node of this axis. Integrating these dimensions into clinical assessment and therapeutic trials will be essential for precision-medicine approaches that protect not only the liver, but also mobility, fracture resistance, renal health, cardiovascular outcomes and long-term functional independence.

## 9. MASLD–Heart Axis

### 9.1. Epidemiological Evidence

The MASLD–heart axis is best viewed as a two-way clinical continuum rather than a chance coexistence of common disorders. Earlier studies used the NAFLD/NASH nomenclature; these populations largely map into present-day MASLD and MASH, although diagnostic criteria are not identical. CVD is the leading cause of death in MASLD, and risk rises with steatohepatitis and, most consistently, fibrosis severity. In Targher’s seminal meta-analysis, imaging- or histology-defined NAFLD predicted incident fatal and non-fatal CVD, with stronger associations in severe disease [171]. Mantovani’s updated analysis likewise showed excess fatal and non-fatal cardiovascular events across large longitudinal cohorts [172]. Outcome-specific evidence is coherent. Alon’s meta-analysis associated NAFLD with myocardial infarction, ischemic stroke, atrial fibrillation, and heart failure [173]. Mantovani’s analysis of 11 cohorts, approximately 11.2 million participants, and 97,716 incident heart-failure cases estimated a 50% higher heart-failure risk after adjustment for conventional risk factors [174]. In a nationwide histology cohort study, 10,422 Swedish adults with biopsy-confirmed NAFLD experienced more major adverse cardiovascular events than matched controls over a median 13.6 years; absolute incidence was 24.3 versus 16.0 per 1000 person-years, and risk increased across histological severity [175].

Collectively, these studies tend to support MASLD as a cardiovascular risk enhancer. Nevertheless, residual confounding by obesity, diabetes, hypertension, kidney disease, and health-care ascertainment prevents equating association with causality. In agreement, genetic analyses reveal that the systemic consequences of liver fat depend on its biological origin. Variants impairing hepatic triglyceride export increase liver fat yet associate with lower coronary risk, whereas variants promoting de novo lipogenesis increase both liver fat and coronary disease [176]. By contrast, greater liver fat consistently raises the risk of cirrhosis and hepatobiliary cancers, irrespective of mechanisms. Therefore, MASLD/SLD may not be intrinsically cardiotoxic, with cardiovascular risk reflecting the pathway driving lipid accumulation, whereas liver and cancer risk invariably scale with hepatic fat burden.

Reverse traffic along the MASLD–heart axis is increasingly recognized. Established coronary disease, atrial fibrillation, and especially heart failure can accelerate hepatic injury through reduced forward flow, venous congestion, hypoxemia, neurohormonal activation, reduced mobility, sarcopenia, and treatment-related metabolic change. In acutely decompensated heart failure, hepatic congestion can distort aminotransferases, stiffness measurements, and fibrosis scores; nevertheless, Valbusa et al. reported that ultrasonographic NAFLD and advanced non-invasive fibrosis identified higher mortality among older patients admitted with acute heart failure [177]. The epidemiological picture is therefore bidirectional but asymmetric: evidence that MASLD precedes CVD is extensive, whereas prospective evidence that incident CVD accelerates MASLD progression is newer and more vulnerable to diagnostic misclassification.

### 9.2. Mechanisms

A shared upstream substrate—visceral adiposity, insulin resistance, T2D, hypertension, atherogenic dyslipidemia, sleep apnea, sedentary behavior, and CKD—initiates both hepatic and cardiac disease. Insulin-resistant adipose tissue releases excess non-esterified fatty acids and inflammatory adipokines; the liver adds de novo lipogenesis and very-low-density lipoprotein export. When disposal is exceeded, toxic lipid species, mitochondrial dysfunction, endoplasmic-reticulum stress, and reactive oxygen species activate hepatocyte injury, Kupffer cells, and stellate-cell fibrogenesis. The resulting liver is not merely a marker: it releases glucose, triglyceride-rich and apolipoprotein-B-containing particles, ceramides, cytokines, procoagulant factors, and altered hepatokines that promote endothelial dysfunction, plaque formation, thrombosis, myocardial lipid deposition, and fibrosis. Authoritative reviews emphasize insulin resistance, systemic inflammation, genetic susceptibility, and gut microbial dysbiosis as intersecting pathways [178,179].

Several mechanisms favor specific cardiac phenotypes. Endothelial dysfunction, oxidative modification of lipoproteins, impaired fibrinolysis, and platelet hyperreactivity accelerate atherosclerosis. Epicardial and perivascular adipose inflammation, autonomic imbalance, and atrial structural remodeling favor atrial fibrillation. Sodium retention, microvascular rarefaction, impaired myocardial energetics, and systemic inflammation promote concentric remodeling, diastolic dysfunction, and heart failure with preserved ejection fraction; ischemia and lipotoxic cardiomyopathy may also contribute to reduced-ejection-fraction disease. Gut-derived endotoxin, trimethylamine-N-oxide, bile-acid signaling, and intestinal permeability provide plausible amplifiers, but human causal evidence remains incomplete. Genetic variants illustrate dissociation: PNPLA3 and TM6SF2 can increase liver injury while producing neutral or lower circulating lipids, whereas GCKR may increase both hepatic fat and cardiometabolic risk. Consequently, liver fat alone is an imperfect surrogate for cardiovascular hazard; fibrosis burden and systemic subclinical chronic inflammation in the context of the accompanying metabolic phenotype are probably more informative.

The heart-to-liver limb includes hemodynamic and behavioral mechanisms. Low cardiac output activates sympathetic and renin–angiotensin–aldosterone systems, worsening insulin resistance and adipose lipolysis. Elevated right-sided filling pressure produces sinusoidal congestion, endothelial injury, centrilobular hypoxia, and ultimately “cardiac cirrhosis”; repeated decompensation may superimpose congestive hepatopathy on metabolic steatosis. Exercise intolerance reduces energy expenditure, while cachexia or sarcopenic obesity impairs glucose disposal. Conversely, portal hypertension and advanced cirrhosis can induce hyperdynamic circulation and cirrhotic cardiomyopathy. Huang and colleagues accordingly describe CVD and MASLD as sharing mechanisms rather than forming a simple linear chain [179]. Clinically, congestion must be reassessed after decongestion before attributing high liver stiffness to fixed MASH fibrosis.

### 9.3. Principles of Treatment

The governing principle is simultaneous reduction of hepatic, cardiovascular, renal, and metabolic risk rather than sequential organ-specific care. Joint EASL–EASD–EASO guidance recommends case-finding for advanced fibrosis in high-risk groups, particularly T2D or obesity with additional metabolic factors, using FIB-4 followed by transient elastography or an equivalent second-line test [10]. Treatment starts with a Mediterranean-style, minimally processed diet; reduced sugar-sweetened beverages and refined carbohydrates; resistance plus aerobic exercise; smoking cessation; adequate sleep; and limited alcohol. Weight loss is dose-responsive: approximately 5% improves steatosis, 7–10% often improves steatohepatitis, and greater sustained loss offers the best chance of fibrosis regression [180]. Exercise and dietary quality remain valuable even without major weight loss.

Cardiovascular prevention should follow absolute-risk and comorbidity guidelines: aggressively control blood pressure, LDL cholesterol, glycaemia, obesity, sleep apnea, and kidney disease. Statins are appropriate and generally safe in compensated MASLD; mildly elevated aminotransferases alone are not a reason to withhold them. In T2D, glucagon-like peptide-1 receptor agonists are attractive when obesity or atherosclerotic CVD predominates, whereas sodium–glucose cotransporter-2 inhibitors are particularly valuable in heart failure or CKD; liver benefits should not be overstated because cardiovascular-outcome trials were not designed around histological MASH. Bariatric/metabolic surgery can produce substantial, durable weight loss in selected non-cirrhotic patients. For non-cirrhotic MASH with significant fibrosis, resmetirom is a liver-directed option where approved: Harrison’s phase 3 MAESTRO-NASH trial demonstrated higher rates of MASH resolution, and at least one-stage fibrosis improvement, alongside reductions in LDL cholesterol, but definitive cardiovascular-outcome evidence is pending [124]. Advanced cirrhosis, decompensated heart failure, and polypharmacy require specialist co-management.

### 9.4. Implications for Precision-Medicine Clinical Practice

Precision practice should phenotype both axes. First, define hepatic stage using a sequential non-invasive pathway rather than aminotransferases alone: FIB-4, then elastography or enhanced liver fibrosis testing, with hepatology referral for discordant results or advanced fibrosis. Interpret age, thrombocytopenia, rhythm, and volume status because they alter test performance. Second, characterize the cardiovascular phenotype: established atherosclerotic disease, heart-failure subtype, atrial fibrillation, CKD, blood pressure, apolipoprotein-B burden, smoking, sleep apnea, and functional capacity. Select therapy by the dominant near-term threat while seeking cross-organ benefit—for example, an SGLT2 inhibitor for MASLD with heart failure, a GLP-1 receptor agonist for obesity and atherosclerotic risk, intensive lipid lowering for high apolipoprotein-B exposure, and resmetirom for eligible F2–F3 MASH. This is risk-based prescribing, not simply treatment of liver fat.

Third, monitoring should use organ-relevant end points: weight and waist trajectory, blood pressure, HbA1c, lipids, estimated glomerular filtration rate, heart-failure symptoms, arrhythmia burden, FIB-4, and liver stiffness at clinically meaningful intervals. A change in steatosis does not guarantee lower cardiovascular risk, and congestion-related stiffness should be repeated when euvolemic. Genotyping is not routine, but PNPLA3, TM6SF2, GCKR, and HSD17B13 may eventually refine prognosis or trial eligibility; current ancestry-linked allele frequencies must not become proxies for care. Multidisciplinary pathways linking primary care, hepatology, cardiology, diabetology, obesity medicine, nutrition, and pharmacy are especially important because therapeutic inertia and fragmented follow-up are modifiable risks. Shared decisions should include treatment burden, adverse effects, frailty, affordability, reproductive considerations, and patient priorities.

### 9.5. Summary

The MASLD–heart axis is a bidirectional cardiometabolic system. MASLD, particularly MASH with fibrosis, marks excess risk of atherosclerotic events, atrial fibrillation, and heart failure; cardiovascular dysfunction can in turn worsen steatosis, inflammation, fibrosis, and the interpretation of liver tests through congestion, hypoperfusion, neurohormonal activation, inactivity, and sarcopenia. Shared upstream drivers explain much, but liver-derived lipoproteins, inflammatory mediators, coagulation factors, and hepatokines plausibly add risk. Clinical care should therefore combine fibrosis staging with formal cardiovascular phenotyping, intensive lifestyle and risk-factor treatment, and comorbidity-directed drugs with demonstrated cardiovascular or hepatic benefit. Precision medicine is achieved by matching treatment to fibrosis stage, cardiovascular phenotype, kidney function, obesity, and patient context—not by treating steatosis in isolation.

## 10. Pathomechanisms of Organ Crosstalk in MASLD

MASLD is a systemic disorder sustained by bidirectional communication between the liver and endocrine system, gut, adipose tissue, brain, kidney, muscle, bone, and heart. Neural pathways, hormones, cytokines, adipokines, hepatokines, myokines, osteokines, bile acids, microbial metabolites, extracellular vesicles, microRNAs, lipid intermediates, and immune signals couple hepatic steatosis, inflammation, and fibrosis to extrahepatic dysfunction [73,181,182,183]. Organ axes are therefore defined by reciprocal information flow and by disease arising when integrated signaling architectures fail [182].

Insulin resistance, adipose dysfunction, intestinal dysbiosis, bile-acid remodeling, endocrine disruption, chronic inflammation, and mitochondrial stress converge into mutually reinforcing networks [76]. The principal communication routes are summarized below.

### 10.1. Extracellular-Vesicle Networks

Extracellular vesicles are membrane-bound particles released into biological fluids that transport proteins, lipids, metabolites, messenger RNAs, microRNAs, and other non-coding RNAs to local or distant cells [184]. In MASLD, vesicles from stressed hepatocytes, dysfunctional adipocytes, macrophages, endothelial and immune cells can reprogram metabolism, inflammatory tone and fibrogenic activation beyond their tissue of origin [181].

The adipose–liver axis exemplifies this endocrine nanovector function. Adipose tissue signals through free fatty acids, cytokines and adipokines, but also through vesicular proteins, lipids and RNAs that influence insulin sensitivity and systemic homeostasis [184]. Insulin regulates sorting and secretion of adipocyte small-extracellular-vesicle microRNAs; accordingly, hyperinsulinemia may remodel this communication route.

In high-fat diet-fed male mice, adipocyte small-extracellular-vesicle microRNA-30a-3p exacerbates hepatocyte lipotoxicity and steatosis [84]. Adipose-tissue macrophage vesicles enriched in miR-155 and miR-34a similarly activate hepatic stellate cells and promote fibrosis in obese male mice, linking adipose inflammation to hepatic injury [85].

The steatotic liver also signals outward through hepatokines, inflammatory and procoagulant mediators, circulating microRNAs and vesicle-associated cargo. In the liver–kidney axis, these signals may contribute to endothelial dysfunction, glomerular injury, tubulointerstitial inflammation and fibrogenesis, although systematic renal histology in MASLD remains limited [181].

### 10.2. Exosomes and Extracellular Vesicles: Targeting and Translation

Exosomes are small extracellular vesicles (EVs) of approximately 50–150 nm. Their proteins, lipids, and nucleic acids can reprogram recipient cells, whereas surface integrins and related receptors contribute to tissue targeting [181,184]. Binding can initiate surface signaling or membrane fusion and cytoplasmic cargo release, supporting long-range adipose–liver, gut–liver, liver–kidney, liver–brain and muscle–liver communication. Lipotoxicity, oxidative stress, hypoxia, inflammation and insulin resistance alter release and composition of EVs. Moreover, intrahepatic exchange among hepatocytes, Kupffer cells, stellate cells, sinusoidal endothelial cells and recruited immune cells can further amplify fibrogenic circuits [85].

Because EV cargo reflects tissue state, EVs are candidate biomarkers of adipose-driven disease, inflammatory MASH, fibrosis, renal vulnerability and systemic metabolic stress [185]. Clinical translation nevertheless requires standardized isolation, cargo measurement, tissue-of-origin assignment and validation against histology, imaging and outcomes [186]. Therapeutic strategies include blocking pathogenic release, modifying cargo or engineering vesicles to deliver protective molecules; these approaches remain investigational [187].

### 10.3. MicroRNAs as Endocrine Regulators

MicroRNAs are short non-coding RNAs that suppress translation or promote messenger-RNA degradation, thereby reshaping recipient-cell phenotypes [188]. When packaged in extracellular vesicles, they become endocrine-like signals that coordinate hepatic lipid metabolism, insulin signaling, inflammation, stellate-cell activation and systemic energy balance [188,189].

Adipocyte- and adipose macrophage-derived vesicles transport microRNAs to hepatocytes and stellate cells [82]. Adipocyte-secreted exosomal miR-34a may promote insulin resistance and obesity [190], whereas microRNA-30a-3p, miR-155 and miR-34a exemplify transfer of adipose stress into steatosis and fibrosis [85].

Conversely, hepatocyte- or stellate-cell-derived microRNAs may act on adipose tissue, kidney, vascular endothelium, immune compartments and brain [178,181]. Pathogenic profiles can enhance de novo lipogenesis, suppress mitochondrial oxidation, intensify inflammation or activate stellate cells; protective profiles may favor lipid oxidation, insulin sensitivity, anti-inflammatory macrophage polarization and matrix resolution [188,191].

Vesicle- or protein-protected microRNAs are stable in circulation and could complement liver stiffness, imaging, adipokines, inflammatory markers, bile-acid profiles and microbiome biomarkers [192,193]. However, tissue origin, disease stage, comorbidities, medication and sample processing confound interpretation; future studies should anchor signatures to precisely defined organ axes, histology and outcomes.

### 10.4. Lipid Droplets, Lipid Buffering, and Overflow

Lipid droplets (LDs) are dynamic organelles that buffer energy excess and protect against lipotoxicity [194]. Their contacts with mitochondria and endoplasmic reticulum coordinate fatty-acid storage and oxidation, membrane synthesis and stress responses. In hepatocytes, disturbed droplet homeostasis increases exposure to saturated fatty acids, ceramides and diacylglycerols, promoting mitochondrial overload, reactive oxygen species, insulin resistance and ballooning [194].

Failure of subcutaneous adipose storage drives persistent non-esterified fatty-acid release and hepatic triglyceride deposition [195]. Adipose insulin resistance sustains lipolysis despite hyperinsulinemia, while hepatic SREBP-1c and ChREBP signaling preserves lipogenesis. When mitochondrial oxidation and very-low-density-lipoprotein export cannot match substrate influx, triglycerides and toxic lipid intermediates accumulate, causing oxidative and endoplasmic-reticulum stress [195].

LDs or their constituent lipids may also move between cells, potentially redistributing overload but also spreading lipotoxic, inflammatory or mitochondrial stress among hepatocytes, macrophages, stellate cells and endothelial cells [194]. In skeletal muscle, myosteatosis impairs mitochondrial function and insulin-stimulated glucose disposal, reinforcing hyperinsulinemia and hepatic de novo lipogenesis [137].

However, the functional significance of LDs in intercellular and interorgan communication remains incompletely defined. LDs are primarily recognized as intracellular organelles responsible for lipid storage, mobilization, and interactions with mitochondria and the endoplasmic reticulum. Although mechanisms of intercellular lipid transfer have been described, the current evidence does not support considering intact LDs as established systemic interorgan messengers alongside circulating hormones, cytokines, lipoproteins, metabolites, or EVs.

### 10.5. Metabolic, Endocrine, and Neural Signals

Additional crosstalk is conveyed by microbial metabolites, bile acids, short-chain fatty acids, amino-acid derivatives, hormones, cytokines, neural signals, and membrane contacts [196,197]. Portal circulation exposes the liver to nutrients, microbial products, bile acids and immunomodulatory metabolites [198]. Diet-induced dysbiosis and barrier failure convert this physiological circuit into an amplifier of steatosis, inflammation and fibrosis.

Increased permeability permits portal transfer of lipopolysaccharide, peptidoglycan and bacterial DNA. Kupffer cells, stellate cells, sinusoidal endothelial cells and hepatocytes sense these ligands through Toll-like receptor 4, Toll-like receptor 9 and nucleotide-binding oligomerization domain-like receptors [54]. MyD88–NF-κB, interferon-regulatory-factor and inflammasome signaling induces tumor necrosis factor, interleukin-1β, interleukin-6 and chemokines, linking barrier failure to steatohepatitis and fibrosis [54,199].

Dysbiosis increases hepatotoxic metabolites and erodes mucosal functions [55]. Endogenous ethanol and acetaldehyde aggravate oxidative injury [60], whereas trimethylamine-N-oxide (TMAO), branched-chain amino-acid (BCAA) derivatives and imidazole propionate impair insulin signaling and favor hepatic lipid accumulation [200]. By contrast, butyrate and propionate can support epithelial metabolism, regulatory T-cell differentiation, glucagon-like peptide-1 secretion and AMP-activated protein kinase activity, although effects are context-dependent [55].

Bile acids provide a parallel signaling layer. Intestinal bacteria convert primary into secondary bile acids that activate farnesoid X receptor (FXR) and Takeda G protein-coupled receptor 5 (TGR5) [89]. Intestinal FXR/FGF-19 signaling restrains hepatic bile-acid synthesis, gluconeogenesis and lipogenesis and supports barrier integrity. In MASLD, bile-acid-pool remodeling may blunt FXR, alter TGR5-dependent energy expenditure and immunity, and increase hydrophobic secondary bile acids such as deoxycholic acid, promoting hepatocyte stress and stellate-cell activation [201].

The gut–liver–kidney axis includes lipopolysaccharide, indoxyl sulfate, p-cresyl sulfate and trimethylamine-N-oxide, which reinforce systemic inflammation, endothelial dysfunction, bile-acid dysregulation and renal fibrogenesis [112,202]. In the liver–brain axis, ammonia, microbial products and inflammatory mediators can escape hepatic clearance, disrupt the blood–brain barrier, activate microglia and impair neuronal function [96].

Growth hormone, insulin-like growth factor-1, prolactin, adrenocorticotropic hormone, cortisol, insulin, thyroid hormones, thyroid-stimulating hormone, estrogens and testosterone regulate hepatic lipid handling, mitochondrial oxidation, de novo lipogenesis, inflammation and fibrogenesis [51,203]. Autonomic and vagal circuits relay hepatic sensory information to central nuclei and return outputs that regulate glucose production, lipid handling and inflammation [96,204].

Membrane contact sites enable rapid ion and lipid exchange between intracellular structures without vesicular transport [205]. Although primarily subcellular, they determine how extracellular cues are integrated with mitochondrial, endoplasmic-reticulum, lipid-droplet, and inflammatory stress.

**Figure 3:**
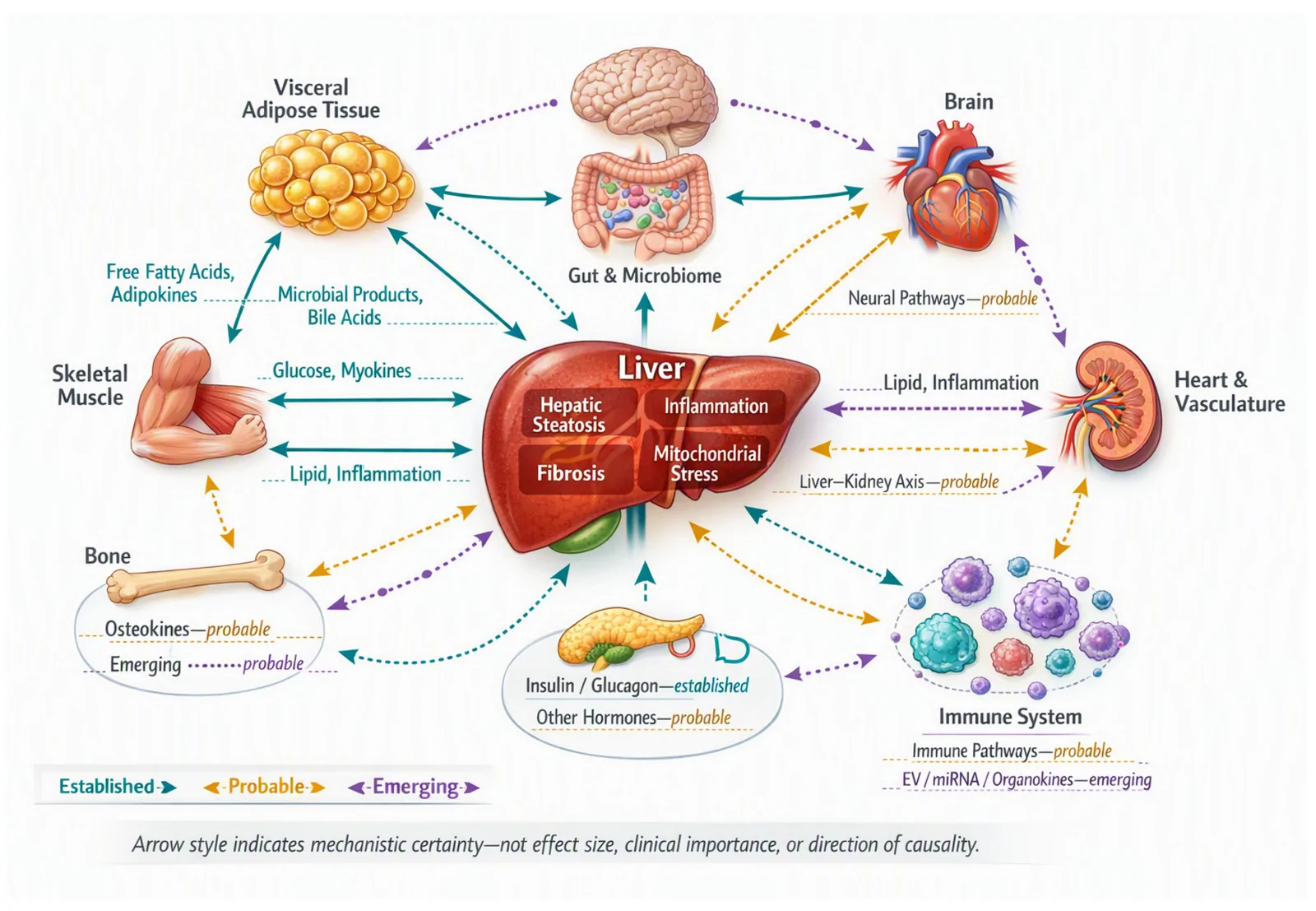
Evidence-tiered model of interorgan crosstalk in metabolic dysfunction-associated steatotic liver disease (MASLD). The liver is positioned at the center of a bidirectional network linking visceral adipose tissue, gut, skeletal muscle, heart and vasculature, kidney, brain, pancreatic/endocrine axis, bone, and immune pathways. Thick solid teal arrows indicate established mechanisms, including free-fatty-acid and adipokine flux, microbial products and bile-acid signaling, glucose and myokine exchange, and lipid- and inflammation-mediated cardio–renal interactions. Dashed amber arrows denote neural, endocrine, pancreatic, and osteokine pathways. Dotted violet arrows represent emerging extracellular vesicle (EV), microRNA (miRNA), and organokine networks. These signals converge on hepatic steatosis, inflammation, mitochondrial stress, and fibrosis. Arrow style indicates the level of mechanistic certainty, not effect size, clinical importance, or direction of causality.

Collectively, vesicular transport, RNA regulation, lipid trafficking, metabolite and bile-acid signaling, endocrine and neural circuits, and immune activation generate the multisystem phenotype of MASLD. Precision hepatology will require identification of the dominant module in each patient—adipose lipid overflow, gut-derived inflammation, endocrine dysfunction, renal vulnerability, neurocognitive susceptibility, or muscle–bone failure—and therapies that target the relevant network rather than the liver alone. However, this framework should be regarded as a precision-medicine hypothesis rather than a clinically validated scoring system. Figure 3 summarizes these mechanisms differentiating established from emerging mechanisms.

## 11. Current Understanding and Broader Insights

### 11.1. Conclusion

MASLD is best understood as a systemic disorder of interorgan communication rather than a liver-confined consequence of lipid excess. The liver functions simultaneously as a sensor, integrator, and source of endocrine, metabolic, microbial, immune, and neural signals. Perturbation of these networks explains why similar degrees of steatosis can be accompanied by markedly different inflammatory activity, fibrosis trajectories, cardiometabolic burden, renal vulnerability, neurocognitive symptoms, and musculoskeletal decline. Across the endocrine–liver, gut–liver, adipose tissue–liver, liver–brain, liver–kidney, MASLD–muscle–bone and MASLD-heart axes, recurrent drivers include insulin resistance, adipose storage failure, dysbiosis, altered bile-acid signaling, endocrine disruption, mitochondrial stress and chronic inflammation. These processes are propagated by EVs, microRNAs, hepatokines, adipokines, myokines, osteokines, cytokines, SCFAs, microbial metabolites, lipid intermediates and autonomic pathways. Their effects are contextual and bidirectional: adaptive signals can buffer metabolic stress early but become self-reinforcing once tissue capacity is exceeded.

This network perspective has direct clinical implications. For example, it may help to differentiate which biomarkers or combinations of measurements are currently clinically actionable, as opposed to investigational biomarkers that require additional research before entering the clinical arena (Table 2).

Adipose failure controls substrate delivery and inflammatory tone; the gut shapes microbial and bile-acid exposure; endocrine organs regulate lipid oxidation, DNL and fibrosis; the kidney, brain, muscle and bone both receive liver-derived signals and feedback on systemic resilience. Accordingly, liver fat alone is an incomplete measure of disease. Precision phenotyping should combine steatosis and fibrosis assessment with cardiometabolic risk, adipose distribution, endocrine context, kidney function, cognitive and sleep symptoms, muscle strength and quality, and bone health. The aim is not to catalogue every abnormal axis, but to identify the dominant mechanisms that determine progression and treatment response in an individual patient.

### 11.2. Research Agenda

While the interpretation that most interorgan axes are bidirectional crosstalk is well supported mechanistically for the gut–liver and adipose tissue–liver axes, much of the evidence for the liver–brain, liver–kidney, and especially the muscle–bone–liver networks remains observational in humans, and the direction of causality is frequently uncertain. This finding supports the necessity of additional mechanistic studies.

The research agenda should first establish longitudinal, deeply phenotyped cohorts spanning steatosis, MASH, advanced fibrosis, cirrhosis, HCC and extrahepatic complications. Repeated measures should integrate imaging, elastography and histology with metabolic and renal indices, cognitive and sleep assessment, body composition, bone density, diet, physical activity and medication exposure. Such designs are required to distinguish early causal signals from consequences of established tissue injury and to determine whether changes in one organ precede, accompany or follow changes in another.

Second, multi-omic studies should connect metagenomics, metabolomics, lipidomics, proteomics, transcriptomics, EV profiling, and single-cell and spatial biology to organ-specific physiology. The priority is to define reproducible crosstalk modules rather than generate additional descriptive signatures. Tissue of origin, target-cell response, disease stage and temporal dynamics must be resolved for candidate microbial metabolites, bile-acid profiles, adipose-derived vesicles, hepatokines, myokines and microRNAs.

Third, experimental models should move beyond single-organ perturbations. Clinically relevant systems must incorporate combinations of obesity, T2D, ageing, sex-hormone change, hypothalamic–pituitary dysfunction, CKD, sarcopenia, dysbiosis and cardiovascular risk. Human organoids, linked microphysiological systems and spatially resolved tissue studies could complement animal models by testing directionality and context dependence across multiple organs.

Fourth, sex, reproductive stage and endocrine status should be embedded prospectively in study design. Men, premenopausal and postmenopausal women, and women with PMOS, formerly known as PCOS, differ in adipose distribution, estrogen and androgen signaling, muscle reserve, bone turnover and fibrosis risk. These variables should inform sampling, thresholds, endpoints and therapeutic priorities rather than be treated as post hoc modifiers.

Fifth, biomarkers should identify clinically actionable endotypes, including adipose-driven, gut-inflammatory, endocrine-associated, fibrosis-dominant, renal-risk, neurocognitive-risk and sarcopenic or osteosarcopenic MASLD. Validation must extend beyond association with steatosis to outcomes that matter: fibrosis progression, liver-related events, CVD, CKD progression, cognitive decline, falls, fractures, frailty, quality of life and mortality.

Sixth, trials should test whether modifying one node restores resilience across the network. Incretin-based therapies, THR-β agonists, bariatric surgery, microbiome-directed interventions, endocrine correction, exercise and adipose-directed strategies should therefore include liver, glycemic, renal, vascular, cognitive, functional and skeletal endpoints. Mechanistic substudies should determine whether extrahepatic improvement mediates histological response or emerges independently.

Lifestyle interventions should likewise be studied as mechanism-based treatments. Dietary quality, fiber, minimally processed plant-derived foods, adequate protein, calcium and vitamin D, aerobic and resistance exercise, and sleep optimization can simultaneously reshape microbiota, adipose inflammation, insulin sensitivity, muscle function, bone loading and hepatic lipid metabolism. Trials should define which combinations benefit specific endotypes and how lean mass and bone are preserved during substantial weight loss.

Ultimately, precision hepatology should match treatment intensity and combination to the dominant crosstalk architecture. Patients with adipose lipid overflow, dysbiosis-driven inflammation, endocrine-mediated steatosis, renal vulnerability, cognitive symptoms, or osteosarcopenia are unlikely to require identical care. Future guidelines should therefore promote integrated cardio–renal–hepatic–metabolic and musculoskeletal pathways. Remission should denote more than reduced liver fat or improved fibrosis: it should encompass durable metabolic resilience, preserved organ function, prevention of extrahepatic complications and better long-term functional health.

## Figures and Tables

**Table 1:** Overview of the endocrine axes involved in MASLD development and progression.

Endocrine Axis	Primary Hormonal Mediators	Key Hepatic Mechanism in MASLD	Clinical Impact/Outcome	References
Hypothalamus–Pituitary–Liver	GH, IGF-1, PRL, ACTH	↓ Mitochondrial oxidation, ↑ Cortisol-driven lipolysis	Rapidly progressive MASH and accelerated fibrosis	[19]
Pancreas–Liver	Insulin	Hyperinsulinemia, ↓ CEACAM1 clearance, ↑ SREBP-1c/DNL	Intracellular triglyceride buildup and severe steatosis	[52]
Thyroid–Liver	T_3_, T_4_, TSH	Downregulated THR-β activity, TSH-R-mediated DNL induction	Reduced lipid clearance, chronic inflammation, advanced steatosis	[36,53]
Gonad–Liver	Estrogen (ER-α), Testosterone	ER-α blocks lipogenic enzymes; hyperandrogenism drives DNL	Postmenopausal/PMOS-associated progression risk, high sexual dimorphism	[50,51]

Abbreviations: ACTH, adrenocorticotropic hormone; CEACAM1, carcinoembryonic antigen-related cell adhesion molecule 1, also known as CD66a; DNL, de novo lipogenesis; ER, estrogen receptor; GH, growth hormone; IGF, insulin-like growth factor; SREBP-1c, sterol regulatory element-binding protein 1c; MASH, metabolic dysfunction-associated steatohepatitis; PMOS, polyendocrine metabolic ovary syndrome (previously known as PCOS); PRL, prolactin; THR-β, thyroid hormone receptor-β; TSH, thyroid-stimulating hormone. ↑ indicates “increased”, ↓ indicates “decreased”.

**Table 2:** Current clinical actionability versus investigational status of biomarkers and multidimensional measurements in MASLD.

Biomarker or Measurement Combination	Current Status	Clinically Supported Use or Implication	Main Limitation or Research Need
FIB-4 (age, AST, ALT, platelet count)	Clinically actionable	First-line fibrosis risk stratification in at-risk adults; a low result can support follow-up in primary care, whereas an elevated or indeterminate result triggers secondary testing or specialist referral.	Age-dependent performance; reduced accuracy in younger adults and acute illness; not a stand-alone diagnosis of MASH or fibrosis stage.
FIB-4 followed by vibration-controlled transient elastography or ELF	Clinically actionable combination	Guideline-supported sequential pathway to rule out or identify advanced fibrosis, determine referral intensity, and inform surveillance and treatment eligibility.	Discordant or borderline results require expert interpretation and, in selected cases, additional imaging or biopsy.
Liver stiffness measurement by transient elastography; magnetic resonance elastography where available	Clinically actionable	Non-invasive staging of fibrosis risk, prognostic enrichment, and longitudinal reassessment; may contribute to identification of non-cirrhotic F2–F3 disease.	Influenced by inflammation, congestion, cholestasis, obesity, technical quality, and platform-specific thresholds.
ELF test	Clinically actionable in selected pathways	Secondary fibrosis assessment when FIB-4 is elevated or indeterminate; useful where elastography is unavailable and for prognostic enrichment.	Cost, availability, and threshold variation; should be interpreted with clinical context and other non-invasive tests.
Imaging evidence of steatosis plus cardiometabolic risk factors	Clinically actionable	Supports MASLD case identification and prompts fibrosis risk stratification and management of obesity, diabetes, dyslipidemia, hypertension, alcohol exposure, and cardiovascular risk.	Steatosis burden alone does not reliably identify MASH or predict fibrosis-related outcomes.
MRI-PDFF or controlled attenuation parameter	Clinically useful, but context-dependent	Quantifies hepatic fat and can monitor change in specialist practice or trials; complements, but does not replace, fibrosis assessment.	Fat reduction is not equivalent to fibrosis regression or improved clinical outcomes; access and cost vary.
F2–F3 fibrosis established by concordant non-invasive tests or biopsy, with exclusion of cirrhosis	Clinically actionable combination	Defines a population for MASH-directed pharmacotherapy where approved and label-concordant, alongside diet and exercise; informs closer fibrosis monitoring.	Exact thresholds and access are jurisdiction-specific; indeterminate or discordant tests may require hepatology review.
HbA1c or fasting glucose, lipid profile, blood pressure, body mass index or waist measures	Clinically actionable	Identifies treatable cardiometabolic drivers and guides diabetes, obesity, lipid, and blood-pressure therapy with cardio–renal–hepatic benefit.	These measures do not stage liver fibrosis and should be combined with liver-specific risk assessment.
Serum creatinine/eGFR plus urinary albumin-to-creatinine ratio	Clinically actionable combination	Detects and stages CKD, refines cardiovascular and medication risk, and guides kidney-protective therapy and integrated cardio–kidney–liver care.	Does not establish that renal injury is caused by MASLD; trends and competing renal causes must be assessed.
Grip strength or chair-stand performance plus muscle mass/quality assessment; DXA and fracture-risk assessment when indicated	Clinically actionable for comorbidity management	Identifies sarcopenia, frailty, and skeletal fragility and guides resistance exercise, nutritional support, fall prevention, and osteoporosis management.	No MASLD-specific universal thresholds; opportunistic CT/MRI myosteatosis metrics and fat-to-muscle ratios remain insufficiently standardized.
TSH with free T4; targeted GH/IGF-1, cortisol, prolactin, or gonadal hormone testing when clinically indicated	Clinically actionable for suspected endocrine disease	Confirms established endocrine disorders whose guideline-based treatment may modify metabolic and hepatic risk; testing should be phenotype-driven rather than universal.	Isolated hormone concentrations are not validated for routine MASLD staging, prognosis, or treatment selection.
Adiponectin, leptin-to-adiponectin ratio, resistin, chemerin, fetuin-A, FGF21, and other adipokines/hepatokines	Investigational	Promising for adipose-driven or hepatokine-defined endotypes and mechanistic treatment-response studies.	Assay heterogeneity, confounding by adiposity and comorbidities, absent standardized cut-offs, and limited evidence that results change routine care.
Microbiome composition, intestinal permeability, bile-acid profiles, LPS-binding protein, soluble CD14, TMAO, imidazole propionate, and short-chain fatty-acid signatures	Investigational	Candidate gut-inflammatory and metabolic endotypes for prognosis or selection of gut-directed interventions.	Strong effects of diet, drugs, geography, sampling, and disease stage; causality, reproducibility, and clinically validated thresholds remain unresolved.
Circulating extracellular vesicles, exosomal cargo, and microRNA panels	Investigational	Potential liquid-biopsy markers of tissue origin, inflammatory MASH, fibrosis, renal vulnerability, and treatment response.	Requires standardized isolation and normalization, tissue-of-origin assignment, multicenter validation, and demonstration of incremental clinical utility.
Lipidomic, metabolomic, proteomic, transcriptomic, genetic, and integrated multi-omic signatures	Investigational	May define reproducible adipose-, gut-, endocrine-, renal-, neurocognitive-, or muscle–bone-dominant endotypes and improve trial enrichment.	High dimensionality, cost, limited portability, uncertain causal interpretation, and lack of prospective evidence that omic-guided decisions improve outcomes.
Myokines and osteokines, including myostatin, irisin, osteocalcin, osteopontin, sclerostin, and FGF23	Investigational for MASLD stratification	Mechanistic candidates for sarcopenic, myosteatotic, or osteosarcopenic endotypes and cross-organ therapeutic studies.	Not standardized for routine MASLD diagnosis, prognosis, or treatment selection; substantial influence from age, kidney function, exercise, and bone disease.
Cognitive, sleep, autonomic, inflammatory, and liver-fibrosis measures combined as a liver–brain panel	Investigational as a MASLD endotype	May identify neurocognitive vulnerability and support multidomain trial endpoints; conventional symptom assessment remains clinically relevant.	No validated MASLD-specific composite or evidence that biomarker-guided neurological surveillance improves outcomes.

Interpretation: “Clinically actionable” denotes a measurement that can presently alter referral, staging, surveillance, treatment of comorbid disease, or eligibility for an approved intervention when used in the appropriate clinical context. “Investigational” denotes a promising mechanistic or prognostic marker that lacks sufficient standardization, validated thresholds, prospective outcome data, or evidence of incremental decision benefit for routine care. Local regulatory approval and professional guidance should govern treatment decisions. Abbreviations: ALT, alanine aminotransferase; AST, aspartate aminotransferase; CKD, chronic kidney disease; DXA, dual-energy X-ray absorptiometry; eGFR, estimated glomerular filtration rate; ELF, enhanced liver fibrosis; FIB-4, fibrosis-4 index; FGF, fibroblast growth factor; HbA1c, glycated hemoglobin; LPS, lipopolysaccharide; MASH, metabolic dysfunction-associated steatohepatitis; MASLD, metabolic dysfunction-associated steatotic liver disease; MRI-PDFF, magnetic resonance imaging proton density fat fraction; TMAO, trimethylamine-N-oxide; TSH, thyroid-stimulating hormone.

## Data Availability

No new data were created or analyzed in this study. Data sharing is not applicable to this article.

## Abbreviations

The following abbreviations are used in this manuscript:

ACC	Acetyl-CoA carboxylase
ACTH	Adrenocorticotropic hormone
AMPK	AMP-activated protein kinase
BCAA	Branched-chain amino acid
CEACAM1	Carcinoembryonic antigen-related cell adhesion molecule 1
ChREBP	Carbohydrate response element-binding protein
CKD	Chronic kidney disease
CPT1A	Carnitine palmitoyltransferase 1A
CVD	cardiovascular disease
DNL	De novo lipogenesis
eGFR	Estimated glomerular filtration rate
EV(s)	Extracellular vesicles
FAS	Fatty-acid synthase
FGF	Fibroblast growth factor
FXR	Farnesoid X receptor
GH	Growth hormone
HCC	Hepatocellular carcinoma
IGF-1	Insulin-like growth factor 1
LD(s)	Lipid droplet(s)
LDL	Low-density lipoprotein
LPS	Lipopolysaccharide
MAFLD	Metabolic dysfunction-associated fatty liver disease
MASH	Metabolic dysfunction-associated steatohepatitis
MASLD	Metabolic dysfunction-associated steatotic liver disease
MyD88	Myeloid differentiation primary response 88
NAFLD	Nonalcoholic fatty liver disease
NASH	Nonalcoholic steatohepatitis
NF-κB	Nuclear factor kappa-light-chain-enhancer of activated B cells
Nrf2	Nuclear factor erythroid 2-related factor 2
PAMPs	Pathogen-associated molecular patterns
PCOS	Polycystic ovary syndrome
PGC-1α	Peroxisome proliferator-activated receptor gamma coactivator 1-α
PI3K	Phosphoinositide 3-kinase
PMOS	Polyendocrine metabolic ovary syndrome
PPARα	Peroxisome proliferator-activated receptor-α
PRL	Prolactin
SCFAs	Short-chain fatty acids
SLD	Steatotic liver disease
SREBP-1c	Sterol regulatory element-binding protein 1c
T2D	Type 2 diabetes
T3	Triiodothyronine
T4	Thyroxine
TGF-β	Transforming growth factor-β
TGR5	Takeda G protein-coupled receptor 5
THR-β	Thyroid hormone receptor-β
TLR4	Toll-like receptor 4
TLR9	Toll-like receptor 9
TMAO	Trimethylamine-N-oxide
TNF	Tumor necrosis factor
TSH	Thyroid-stimulating hormone
TSH-R	Thyroid-stimulating hormone receptor
VLDL	Very-low-density lipoprotein
